# Generating CRISPR-edited clonal lines of cultured *Drosophila* S2 cells

**DOI:** 10.1093/biomethods/bpae059

**Published:** 2024-08-17

**Authors:** John M Ryniawec, Anastasia Amoiroglou, Gregory C Rogers

**Affiliations:** Department of Cellular and Molecular Medicine, University of Arizona Cancer Center, University of Arizona, Tucson, AZ 85724, United States; Department of Cellular and Molecular Medicine, University of Arizona Cancer Center, University of Arizona, Tucson, AZ 85724, United States; Department of Cellular and Molecular Medicine, University of Arizona Cancer Center, University of Arizona, Tucson, AZ 85724, United States

**Keywords:** *Drosophila*, CRISPR genome editing, Cas9, Schneider S2 cells, centrosome, centriole, microtubule, Polo-like kinase 4/Plk4

## Abstract

CRISPR/Cas9 genome editing is a pervasive research tool due to its relative ease of use. However, some systems are not amenable to generating edited clones due to genomic complexity and/or difficulty in establishing clonal lines. For example, *Drosophila* Schneider 2 (S2) cells possess a segmental aneuploid genome and are challenging to single-cell select. Here, we describe a streamlined CRISPR/Cas9 methodology for knock-in and knock-out experiments in S2 cells, whereby an antibiotic resistance gene is inserted in-frame with the coding region of a gene-of-interest. By using selectable markers, we have improved the ease and efficiency for the positive selection of null cells using antibiotic selection in feeder layers followed by cell expansion to generate clonal lines. Using this method, we generated the first acentrosomal S2 cell lines by knocking-out centriole genes Polo-like Kinase 4/Plk4 or Ana2 as proof of concept. These strategies for generating gene-edited clonal lines will add to the collection of CRISPR tools available for cultured *Drosophila* cells by making CRISPR more practical and therefore improving gene function studies.

## Introduction

Genome-editing technologies enable precise manipulation of DNA, offering researchers a high degree of control over an organism’s genome. To achieve this, CRISPR/Cas9 methods have been developed and tailored for use in many different model organisms, both *in vivo* and in cell culture. The CRISPR/Cas9 system was originally adapted from bacteria and takes advantage of the Cas9 endonuclease [1]. Cas9 induces double-stranded breaks (DSBs) at protospacer adjacent motifs (PAM), short sequences that are found throughout the genome (typically NGG). Cas9 is directed toward specific genomic loci by a guide RNA (gRNA), which contains 18–20 bp of sequence homology to regions directly upstream of PAM sites [2]. Together with a common scaffolding RNA molecule, the unique gRNA complex interacts with Cas9 and provides specificity for Cas9’s endonuclease activity [2]. Currently, CRISPR/Cas9 technologies use a single gRNA (sgRNA) where the unique gRNA is fused to the common scaffold RNA on a single polynucleotide strand [3].

After cleavage by Cas9, the DSB recruits the cell’s endogenous repair machinery, which can be manipulated to edit the genome. Many knock-out schemes rely on error-prone end-joining mechanisms to generate insertions and deletions (indels) that induce frameshifts and create premature termination (stop) codons [3]. Conversely, knock-in techniques rely on homologous recombination (HR)-mediated approaches to insert specific sequences during genome repair [4]. Regardless, genome editing approaches generally rely on these events occurring on all copies of the gene to induce an efficient knock-out or knock-in. Furthermore, in the case of cell culture, these techniques require the selection of single clones to generate tractable experimental systems. Therefore, CRISPR/Cas9 genome editing is still difficult in models with complex genomes and cell lines that are difficult to select clonal lines.

Historically, *Drosophila* has been an attractive genetic model system as its genome has much less functional redundancy compared with mammalian systems, making it easier to generate loss-of-function phenotypes. Not surprisingly, CRISPR/Cas9 has been adapted and widely used for whole flies and cell culture [4–13], including knock-in of N- or C-terminals tags in isolated ovarian somatic cells [5], Kc167 cells [13], as well as S2R+ [6] and its precursor Schneider 2 (S2) cells [4]. The S2 line is a useful model system for basic cellular research because S2 cells are easy and relatively inexpensive to culture and maintain, highly sensitive to RNAi, and easy to image through microscopy methods [14]. However, S2R+ and S2 cells possess an aneuploid genome with approximately two X chromosomes and four copies of each of the two major autosomes [15]. In addition, the S2 genome is segmental, containing anywhere from one to greater than five copies of an individual gene [15]. Thus, it can be challenging to ensure efficient editing at all gene loci.

Despite this, both homology-directed repair (HDR) and indel methods have been used to edit S2R+ cell [9, 12] and S2 cell [8] genomes. CRISPR knock-in methods to tag endogenous genes with fluorescent markers use polymerase chain reaction (PCR)-amplification, immunofluorescence, or Western blot to verify the insertion of fluorescent tags [4]. Depending on the nature of the study, the insertion of a tag to only one locus can be sufficient. Conversely, knock-out studies rely on editing all alleles of the target gene and generally necessitate generating cell populations derived from individual clones. Previous methods in S2R+ cells have co-transfected Cas9-expressing cells with sgRNA and green fluorescent protein (GFP) expression constructs then used fluorescence-activated cell sorting (FACS) to isolate single-positive transfectants into individual wells of a 96-well plate [9, 12]. The propagated clonal populations were then screened with high-resolution melting assays and Sanger sequencing to validate these short mutations in all alleles [12].

An optimized method combines these strategies to generate gene knockouts through the insertion of an antibiotic-resistance gene and sgRNA expression cassettes into the coding region of the gene-of-interest [9]. Expression of Cas9 and four sgRNA increases the likelihood that all copies of the gene will be cut by Cas9 and, thus, subject to HDR with the introduced repair template [9]. Resultant clones can then be screened through PCR amplification of the region, offering a faster and more cost-effective method to screen knockouts [9]. Notably, constitutive expression of Cas9 and the four unique sgRNA within this system increases the likelihood of off-target effects, and relies on cloning the unique HDR templates and tandem sgRNA for each project.

Here, we present an alternative “knock-in to knock-out” strategy and describe methods to adapt these versatile tools to other protocols. Of note, developing this method was necessitated by our inability to replicate aspects of previous CRISPR methods in S2 cells (described below in more detail). Similar to Xia *et al*. [9], our rationale was to insert an antibiotic resistance gene in-frame with the target gene allowing us to select for gene-specific editing and use PCR-based genotyping to detect knock-in of the antibiotic-resistance gene during secondary screening. Additionally, similar to Kunzelmann *et al*. [4], this method limits cloning by using PCR amplification to generate the homologous repair (HR) template and sgRNA. Furthermore, our streamlined CRISPR system can be used in any cultured *Drosophila* cell line that can be transfected, providing another tool in the *Drosophila* molecular toolbox. Finally, we provide two proof of concepts for our “knock-in to knock-out” method to generate null lines via HDR.

## Materials and methods

### Cell culture and transfection

*Drosophila* S2 cells (Invitrogen) were cultured in Sf-900II SFM media (Life Technologies) [14]. Double-stranded RNA (dsRNA) and DNA plasmids were transfected into S2 cells by nucleofection [16]. Briefly, ∼5 × 10^6^ cells were pelleted by centrifugation. Cell pellet was resuspended in 100 µl of transfection solution (5 mM KCl, 15 mM MgCl_2_, 120 mM sodium phosphate, and 50 mM D-mannitol, pH 7.2) containing 2 µg of purified plasmid, and/or purified PCR-amplified sgRNA and HR template, transferred to a cuvette (2 mm gap size), and then electroporated using a Nucleofector 2b (Lonza), program G-030. Transfected cells were diluted immediately with 0.4 ml SF-900 II medium and plated in a 6-well cell-culture plate containing 1 ml of fresh media. Cells were allowed 24 h to recover before additional handling. Expression of all constructs was induced by addition of 0.2–0.7 mM CuSO_4_ to the culture medium. Slimb RNAi was performed by applying 10 µg of Slimb dsRNA to culture medium per day for 7 days. A complete list of reagents and primer sequences is shown in Supplementary Table S1.

For CRISPR editing, ParCas9 cells were first RNAi-treated with 10 µg of mus308 and 10 μg of Lig4 dsRNA [4] per day for a total of 10 days and then transfected with two PCR products (1 µg of each): (i) U6: sgRNA-polyA amplified with primers U6-F (CTCCCGGAGAC GGTCACAGCATCTGTTCGACTTGCAGCCTGAAATACG) and polyA-R (GCTTGTCTGCT CCCGGCATCCGCGGCGTATCACGAGGCCCTTTCG) (664 bp), and (ii) Plk4-neo HR template amplified with either primers Plk4-neo HR- F (AAGACATTCCAAGTGGCTCAGTAA GTCTACAGAAAAAAGGCTAGCTATGTTAATTGAACAAGATGGATTGC) and R (TCCGTC CGACTGCCGCCTTCCAGCACACCCACCTCAATTGTTTCTCCAAATCAGAAGAACTCGTCAAGAAGGCG) (715 bp) or Ana2-neo HR template amplified with primers Ana2-neo HR-F (CAAATACGCTCCAAATGTTTGTTCCCGAAACGGAGGATATGCTGCCCAGAATTGAACAAGATGGATTGC) and R (GTGGGCCCTATAATCTCATTGGTGTGGCCCATGGGCACT GCTGCACTCGGCTCAGAAGAACTCGTCAAGAAGGCG) (715 bp).

Limiting dilution was used for monoclonal line isolation and expansion. Confluent CRISPR-edited cell cultures were transferred to a 1 ml tube and hand-pipetted repeatedly to break up any cell aggregates. Cells were then diluted 1:100 in serum-free media and cell count was measured using a hemocytometer. Next, cells were serially diluted in serum-free media to a cell solution of <500 cells/ml. Depending on the antibiotic used in the selection step, cells were seeded at different numbers in 96-well plates containing 0.5 × 10^6^ antibiotic-sensitive wild-type S2 cells (total volume 100 µl/well). For hygromycin selection, cells were seeded at 5, 35, and 50 cells/well (three 96-well plates per dilution for a total of nine plates). For neomycin selection, cells were seeded at 1, 2, and 5 cells/well (three 96-well plates per dilution for a total of nine plates). Although some wells will grow more than one single colony (and these are avoided), we empirically determined that these dilutions maximize the chances of producing a single colony per well. In the case of hygromycin selection, more cells need to be seeded, likely due to the toxicity of the antibiotic. After 5 days, 100 µl of media containing antibiotic was added to each well (hygromycin 0.25 mg/ml final and G418 1 mg/ml final). After 3 weeks, single colonies should appear and when they fill approximately 25% of the surface area of the well, cells were resuspended and plated into a single well of a 48-well plate with 100 μl of fresh media and no antibiotic. Once cells were confluent, they were resuspended and transferred to a single well in a 24-well plate with 200 μl of fresh media. Once cells were confluent, they were resuspended for secondary screening, and positive cells were further expanded into 6-well plates and then into T25 cm^2^ flasks for expansion and freezing.

### Plasmid construction

3xFLAG-NLS-*spCas9* (human codon-optimized) was synthesized (Twist Biosciences) with flanking KpnI and EcoRI restriction sites, subcloned directionally into the multicloning site of pMT/V5-HisC vector (Invitrogen), and under control of the inducible metallothionein promoter. pMT: 3xFLAG-NLS-Cas9-polyA was excised from pMT/V5-HisC by HindIII digestion and subcloned into pCoHygro (Invitrogen), where a hygromycin-resistance gene is under the *copia* promoter (pHygroCas9). Plk4 and Ana2 gRNA sequences were determined using www.targetfinder.flycrispr.neuro.brown.edu. U6: sgRNA-polyT/polyA cassette was PCR amplified from pAc-sgRNA-Cas9 (Addgene #49330) using gRNA cassette cloning primers 1 (TCCCGGAGACGGTCACAGCATCTGTTCGACTTGCAGCCTGAAATACG) and 2 (GCTTGTCTGCTCCCGGCATCCGCGGCGTATCACGAGGCCCTTTCG (664 bp product) and was subcloned into pHygroCas9 using a PCR-based method. Similarly, a 20 bp Plk4 or Ana2 gRNA was inserted into the gRNA position of the cassette using a PCR-based method using primers Plk4 gRNA-F (GGTATGTTTTCCTCAATACTTCGGGTCTTCGTCCGATTGTGACG TTTTAGAGCTAGAAATAGC) and gRNA-R (GCTATTTCTAGCTCTAAAACGTCACAATCGGA CGAAGACCCGAAGTATTGAGGAAAACATACC) or Ana2 gRNA-F (GGTATGTTTTCCTC AATACTTCGTGCTGCCCAGACTAGCGCCCGTTTTAGAGCTAGAAATAGC) and gRNA-R (GCTATTTCTAGCTCTAAAACGGGCGCTAGTCTGGGCAGCACGAAGTATTGAGGAAA ACATACC) and sequenced using gRNA seq primer (GCAGAGGGTTCTTAAGACC). Two different sources of DH5α competent *Escherichia coli* were used for cloning (Supplementary Table S1). DNA sequences of pHygroCas9 and pHygroCas9-U6gRNA are shown in Supplementary Table S2.

### Immunoblotting

Cells were lysed in PBS-Triton X-100 (0.1%) and protein concentration was determined by Bradford protein assay (BioRad; manufacturer’s instructions) followed by the addition of sodium dodecyl-sulfate polyacrylamide gel electrophoresis (SDS–PAGE) loading buffer. Extracts were boiled for 5 min and stored at −20°C. Samples of equal total protein were resolved by SDS–PAGE, blotted, probed with primary and secondary antibodies, and developed with SuperSignal West Dura Extended Duration Substrate (Thermo Fisher, 34075) using X-ray film. Primary antibodies used for Western blotting include rabbit anti-Ana2, 1:1000 [16]; mouse anti-FLAG monoclonal M2, 1:1000 (Millipore Sigma, F1804), and mouse anti-α-tubulin monoclonal DM1A, 1:1000 (Fisher, PI62204). HRP-conjugated Goat anti-mouse, 1:3000 (Millipore Sigma, 12-349) and anti-rabbit, 1:3000 (Millipore Sigma, 12-348) secondary antibodies were used for detection.

### Immunofluorescence microscopy

S2 cells were spread on concanavalin A-coated glass bottom plates and fixed in ice cold methanol at −20°C for 15 min. Cells were washed with PBS, 0.1% Triton X-100 and blocked in IF blocking buffer (5% normal goat serum in PBS, 0.1% Triton X-100) for 30 min at room temperature. Primary antibodies were diluted in IF blocking buffer and slides were incubated overnight at 4°C. Antibodies were used at the following dilutions: rabbit anti-Plp (1:3000) [17], chicken anti-Cep97 (1:1000) [18], and rat anti-Asl (1:1000) [19]. Slides were washed 3 times with PBS, 0.1% Triton X-100. Secondary antibodies and 3.2 μM Hoechst 33342 (Thermo Fisher Scientific) were diluted in IF blocking buffer and slides were incubated for 30 min. Secondary antibodies were used at the following dilutions: Donkey anti-host animal AlexaFluor 488 (Thermo Fisher Scientific, 1:1500), anti-host animal Rhodamine Red-X (Jackson Immunoresearch, 1:1500), and anti-host animal AlexaFluor Plus 647 (Thermo Fisher Scientific, 1:1500). Slides were then washed 3 times with PBS, 0.1% Triton X-100 and mounted with homemade mounting medium (PBS, 90% glycerol, and 0.1 M propyl gallate) for deconvolution microscopy.

### Microscopy

Phase contrast microscopy of colonies was performed using Zeiss Axiovert 200 with a 20× objective. Spinning disk confocal microscopy was performed using a Yokogawa CSU-W1 spinning disk mounted on a Nikon Ti-2 Eclipse microscope equipped with a sCMOS Kinetix camera (Photometrics) and a Nikon 100× silicone-immersion objective (NA 1.35). Super-resolution microscopy was performed on the Nikon Yokogawa CSU-W1 SoRa spinning disk using the 100× silicone-immersion objective with 2.8× zoom. Microscope control was performed with the Elements software package (Nikon).

### RT-PCR

S2 cells were treated with lig4 and mus308 dsRNA for 7 days. RNA was extracted using the Qiagen RNeasy Mini Kit (Qiagen, #75144) and treated with RNase-Free DNase to remove any genomic contaminants (Qiagen, #79254). Concentration and purity were determined using a BioDrop Duo Spectrophotometer (Biochrom, UK). RT-PCR was then performed according to the SuperScript III One-Step system protocol (Invitrogen, #12574-018), using 10 ng (lig4) or 250 ng (Rp49) of total RNA per reaction. Reverse transcription was carried out at 55°C and then 40 PCR cycles were performed on the resultant cDNA using gene-specific primers with an annealing temperature of 62°C. Total reactions were run on an agarose gel for analysis. Primers used: lig4-F: (5′-CGGCTCATCCTTCAACAGCCACGC-3′) and lig4-R: (5′-GGAAGTAGGA TGCCTTCGCGATGGC-3′) or Rp49-For (5′-AT CCGCCCAGCATACA GG-3′), and Rp49-Rev (5′-CTCGTTCTCTTGAGAACGCAG-3′).

### Statistical analyses

The following statistical test was performed in GraphPad Prism version 10 (GraphPad): 2-tailed ANOVA analysis with Tukey’s *t*-test (Figs 3B, 4C, and 5C) and Student’s *t*-test (Supplementary Fig. S2).

## Results

### Design of the inducible CRISPR constructs in *Drosophila* S2 cells

Our goal was to design a reliable, efficient, and inexpensive CRISPR method for S2 cells that can be easily adapted for both knock-in and knock-out of any gene. Our method involves three main components: (i) a selectable S2 cell line with inducible Cas9 expression, (ii) an sgRNA construct, and (iii) an HR template. These components can be used in different combinations to achieve indel or HDR-mediated editing. For HDR, we generate stable, hygromycin-resistant cells with inducible expression of Cas9 and transfect them with PCR-amplified fragments encoding a sgRNA expression cassette and HR template. For introducing indels, we use an all-in-one plasmid system containing a hygromycin-resistance gene, a sgRNA expression cassette, and an inducible Cas9 construct.

**Figure 1.**
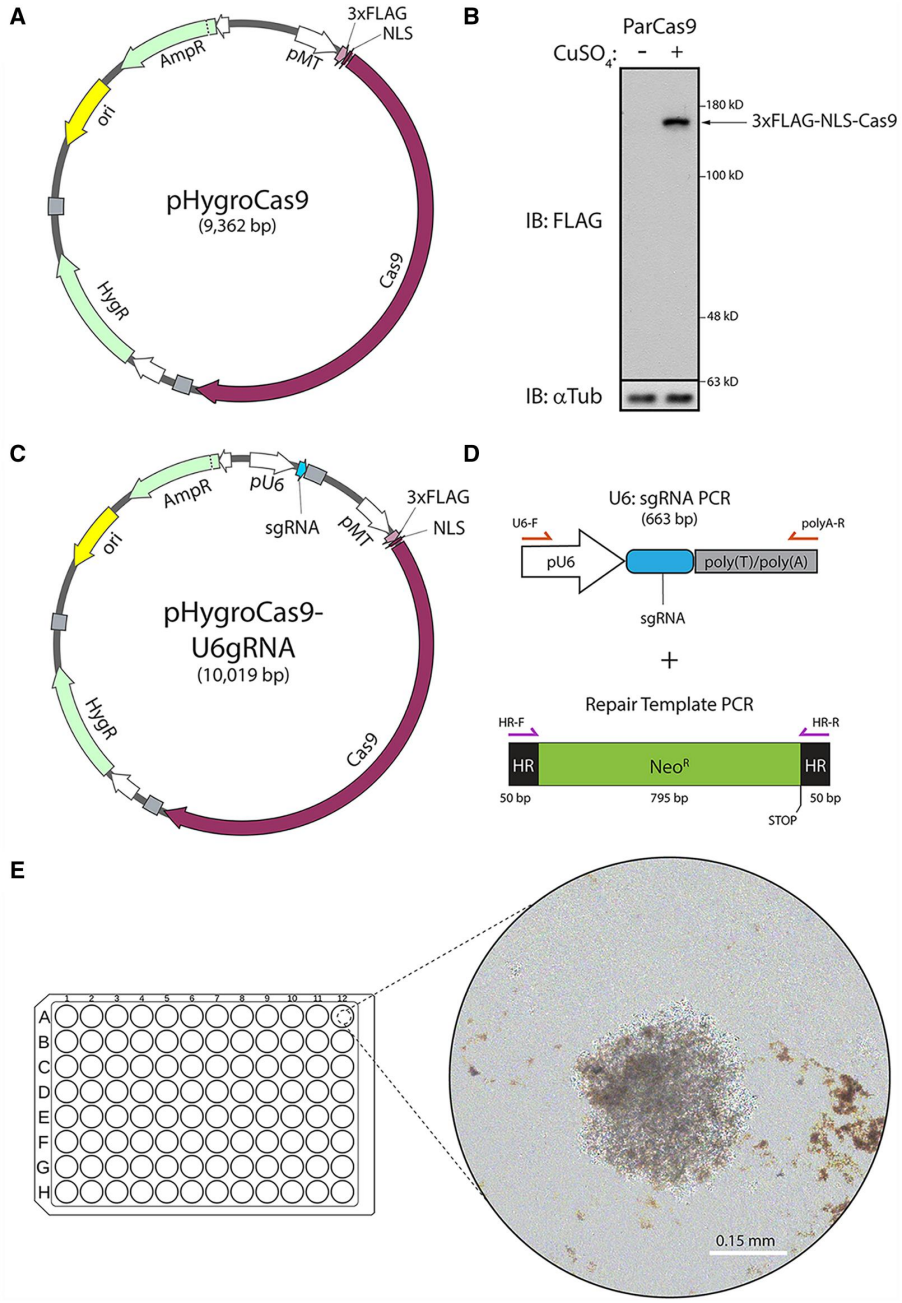
Reagent preparation for generating CRISPR-edited clonal *Drosophila* S2 cell lines. (A) Schematic of the Cas9 expression plasmid (pHygroCas9) containing the hygromycin-resistance gene (Hyg^R^) and 3xFLAG-NLS-Cas9 under control of the inducible metallothionein promoter (pMT). NLS, nuclear localization signal. pHygroCas9 was used to generate the stable pMT : 3xFLAG-Cas9 S2 cell line (referred to as parental line ParCas9). (B) Anti-FLAG immunoblot of a stable ParCas9 cell lysate recognizes transgenic 3xFLAG-NLS-Cas9. Expression was induced by the addition of 0.3 mM CuSO_4_ to the culture medium for 24 h (right lane). Anti-α-tubulin (αTub) was used as a loading control. (C) Schematic of the pHygroCas9-U6gRNA co-expression plasmid containing inducible pMT: 3xFLAG-NLS-Cas9 as well as constitutive U6 promoter (pU6)-driven gRNA and SV40 poly(T)/poly(A) signal. (D) Schematics for the co-transfection of PCR amplified fragments sgRNA cassette (pU6: sgRNA-polyT/polyA) and HR template. In this example, the repair template encodes the neomycin-resistance gene (Neo^R^) containing a stop codon and a frameshift mutation and flanked by 50 nucleotides of HRs for Cas9-mediated homologous recombination. PCR-amplified gRNA and repair template are co-transfected into the ParCas9 cell line and induced to express 3xFLAG-NLS-Cas9. (E) Example of a neomycin-resistant single colony of S2 cells grown under selection in a 96-well plate. Phase contrast image was acquired using a 20× objective.

We began by constructing a hygromycin-resistant, inducible Cas9 expression plasmid (pHygroCas9) for use in S2 cells. First, Cas9 was synthesized with N-terminal 3xFLAG and nuclear localization sequences (NLSs) and cloned into pMT/V5-HisC under the control of the metallothionine (MT) promoter, allowing inducible expression of Cas9 by the addition of CuSO_4_ to the cell culture media. The entire pMT-Cas9-polyA cassette was then subcloned into the backbone of pCoHygro to ensure constitutive expression of the Hygromycin-resistance gene (Hygro^R^) (Fig. 1A). Previously, it was shown that CRISPR editing efficiency is increased in cultured fly cells stably expressing Cas9 [4]. Therefore, we transfected S2 cells with pHygroCas9 and selected for a stably transfected population with hygromycin that was then maintained through passage in hygromycin-containing media. Expression of Cas9 was confirmed in the parental Cas9 (ParCas9) line by immunoblotting for FLAG after induction by CuSO_4_ (Fig. 1B). ParCas9 cells can then be transfected with the desired sgRNA for targeted genome editing.

In parallel, we generated an all-in-one plasmid containing pHygroCas9 and U6 promoter-driven expression of the sgRNA cassette (Fig. 1C). This plasmid allows for antibiotic selection of cells constitutively expressing sgRNA and inducible expression of Cas9. Therefore, Cas9-mediated cleavage of the genome is limited to how long Cas9 is expressed in these cell lines and can be extended when needed. Furthermore, the pHygroCas9-pU6sgRNA can be customized by changing the desired 20-mer gRNA into the existing sgRNA backbone using PCR, essentially designing a longer primer and following a standard site-directed mutagenesis method.

To design the sgRNA, we identified potential sequence using the Fly CRISPR targetfinder web tool (targetfinder.flycrispr.neuro.brown.edu) [20] which generates a list of candidate gRNA sequences upstream of PAM sites from a user-supplied genomic sequence, and then evaluates the candidate sequences for possible off-target sites. Design stringency is user-designated and we selected guide sequences with (i) a length of 20 nt, (ii) a strict “NGG” PAM motif, (iii) and the fewest off-target sites. We note that S2 cells exhibit segmental aneuploidy with a male genotype that is different from the reference *Drosophila* genome [15]. These sequences were then cloned into the pHygroCas9-U6sgRNA plasmid using PCR. While these plasmids can be transfected into cells, they are also used as templates to PCR amplify sgRNA cassettes containing the pU6: sgRNA-polyT/polyA for transfection into ParCas9 cells (Fig. 1D). Conversely, gene-specific sgRNA can be directly PCR amplified from a U6sgRNA template to eliminate the need for cloning (see Methods in Kunzelmann *et al*. [4] for primer and PCR details). Note: for indel generation, we design the sgRNA to target coding regions of the gene-of-interest, whereas for HDR knock-in or knock-out, the sgRNA is not limited to the coding region.

Finally, HDR-mediated knock-in requires the synthesis of an HR template used to introduce mutations or tags into the endogenous gene loci. A major advantage of this approach is to circumvent the use of transgenes that can cause non-physiological phenotypes due to overexpression. When designing the sgRNA, we select genomic regions near the site that will be edited (we have had success by limiting our sgRNA to ∼30 bp around the editing site). The HR template is then designed with homology toward the gene-of-interest flanking the intended insert but lacking the gRNA sequence. For our knock-in to knock-out strategy, we designed an HR template that contains a neomycin-resistance gene (Neo^R^) in-frame with the coding region of our gene-of-interest (Fig. 1D). The HR template contains homology arms flanking the Neo^R^ gene ensuring that the Neo^R^ gene is in-frame with the coding region of the gene-of-interest (50 bp upstream and 50 bp downstream). The Neo^R^ gene itself lacks its own promoter or start codon, thus preventing expression upon random integration, but contains a stop codon (Fig. 1D). Additionally, the homology arms were designed so that repair would create a frameshift immediately after the stop codon. This should prevent spurious expression of the gene-of-interest due to stop codon read-through (refer to Figs 5A, 6A, and 6D for an example of how the HR template is applied). Primers containing these homology arms were then used to PCR amplify the Neo^R^ gene for transfection into cells (Fig. 1D). Cells can then be selected with the antibiotic G418 to select for neomycin resistance.

The final hurdle in generating clonal lines is single-cell selection. S2 cells are semi-adherent and, in our hands, will not grow from single cells with either the addition of serum or conditioned media, thus, neither limiting dilution nor flow sorting are reproducible methods to generate clones in this way. To bypass these limitations, we found that plating limiting dilutions of cells into a feeder layer of antibiotic-sensitive wild-type S2 cells allowed the expansion of CRISPR cell lines. The feeder layer can then be eliminated with the addition of antibiotics, leaving individual colonies derived from the CRISPR clones (Fig. 1E). Cells are then expanded and screened for the desired genomic edit.

### Three methods for efficient gene editing in *Drosophila* S2 cells

**Figure 2.**
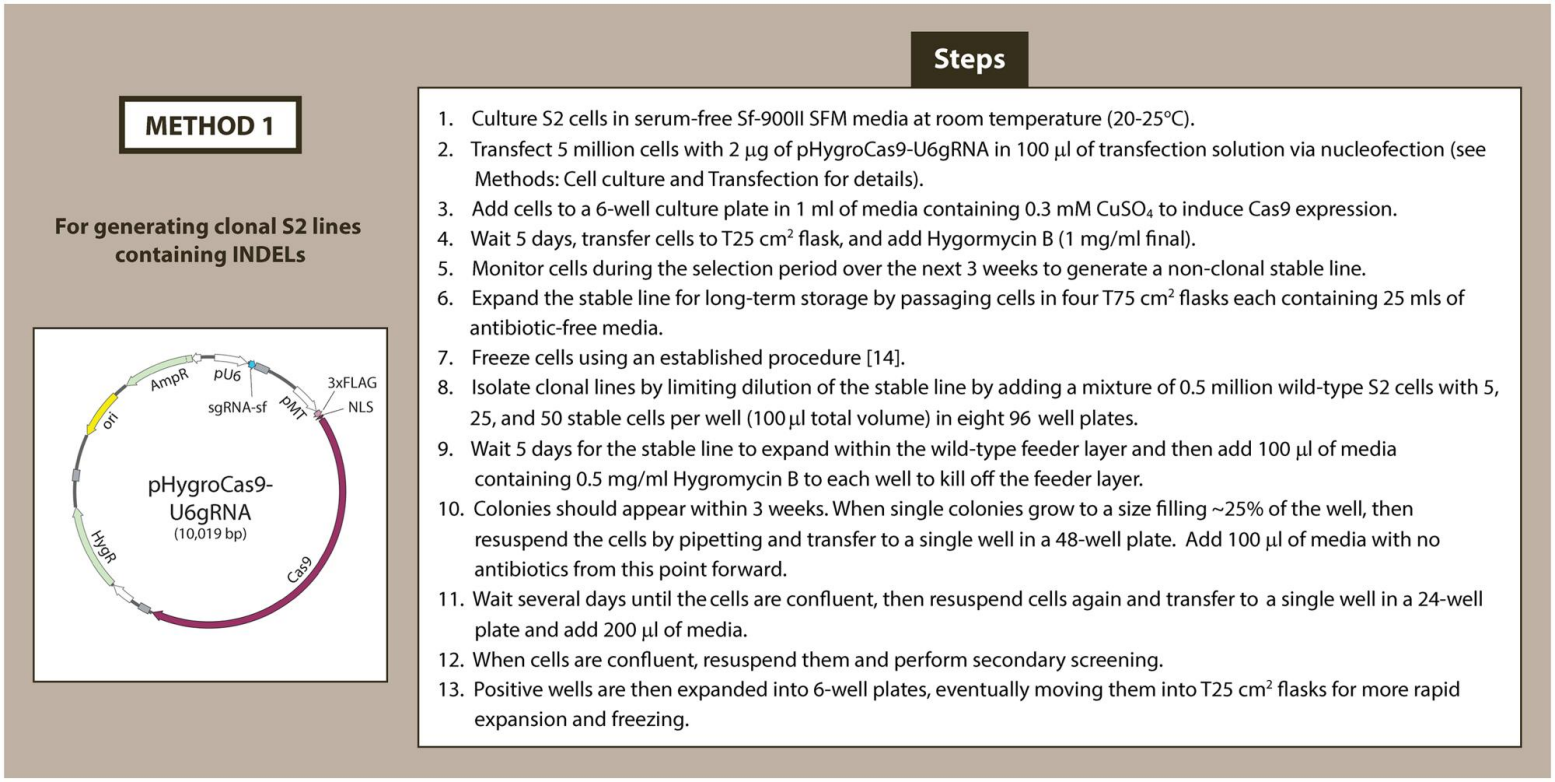
Outline of sequential steps for CRISPR–Cas9 indel generation in cultured S2 cells. This method uses small insertions–deletions (indels) to generate clonal null mutant S2 cell lines by simple transfection of the pHygroCas9-U6sgRNA plasmid.

Using combinations of these tools, our CRISPR system can be used to generate clonal lines using three different methods of editing: (i) introducing indels, (ii) HDR-mediated knock-ins, and (iii) HDR-mediated null lines via neomycin-resistance gene knock-in. The first method generates indels (Fig. 2). The pHygroCas9-U6sgRNA plasmid which contains a sgRNA complementary to the target sequence is transfected into wild-type S2 cells. After 24 h of cell recovery, we induce Cas9 expression using 0.3 µM of CuSO_4_ for 5 days. Cells containing the pHygroCas9-U6sgRNA plasmid are then selected with 1 mg/ml hygromycin to make non-clonal lines. Single cells are isolated by plating limiting dilutions of cells into 96-well plates containing a feeder layer of 0.5 × 10^6^ wild-type S2 cells. After 5 days, hygromycin (0.25 mg/ml final) is added to eliminate the feeder layer. We found that plating at 5, 25, and 50 cells/well results in 10% of wells with single colonies that can be further expanded and screened (for more details, refer to Materials and methods section). Wells that contain more than one colony are avoided. Note: Our method assumes that a clear single round colony originates from a single cell. Clonal lines can then be screened by Western blot or Sanger sequencing as described in previous methods [12]. This method is relatively fast, with clonal lines generated after only 2–3 weeks. However, S2 cells contain greater than two copies of most genes, thus the random nature of indels has a low chance of knocking out all copies of the gene-of-interest [15].

**Figure 3.**
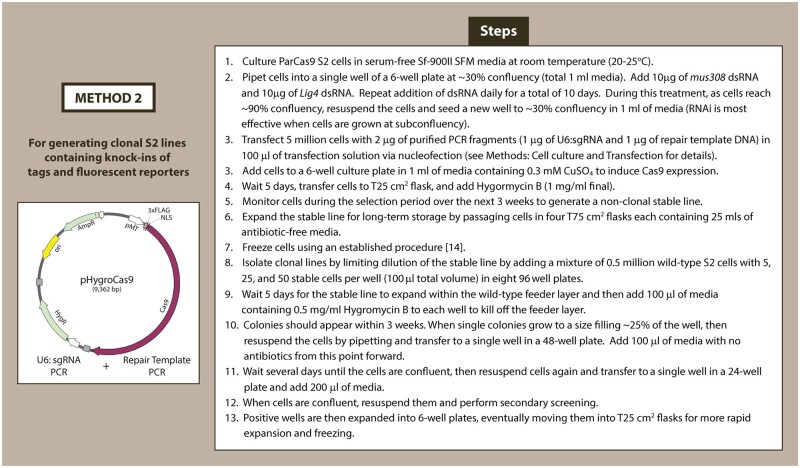
Outline of sequential steps for CRISPR–Cas9 knock-in via HDR in cultured S2 cells. This method describes a gene knock-in strategy in the parental ParCas9 line stably transfected with the pHygroCas9 vector.

The second method allows for mutating endogenous genes or knock-in of tags and fluorescent reporters to the gene-of-interest using HDR (Fig. 3). Similar to previous CRISPR approaches in S2 cells, we first use RNAi to knockdown essential components of alternative DSB repair pathways ensuring that HR is the predominant mechanism to repair Cas9-induced DSB [4]. ParCas9 cells were treated with dsRNA toward DNA Ligase 4 (Lig4; required for non-homologous end joining) and DNA Polymerase Theta (mus308/polQ; required for microhomology-mediated end-joining) for 10 days [4]. We confirmed RNAi knockdown of Lig4 using RT-PCR in the double RNAi-treated cells; however, we were unable to amplify mus308 (Supplementary Fig. S1A). Lig4- and mus308-depleted ParCas9 cells are then transfected with PCR-amplified sgRNA cassette and HR-template containing the tag or appropriate mutations. After 24 h, Cas9 expression is induced with 0.3 µM CuSO_4_ for 5 days and cells are selected as in Method 1. Cells can then be screened with immunofluorescence, Western blot, and PCR amplification of the tag of interest.

**Figure 4.**
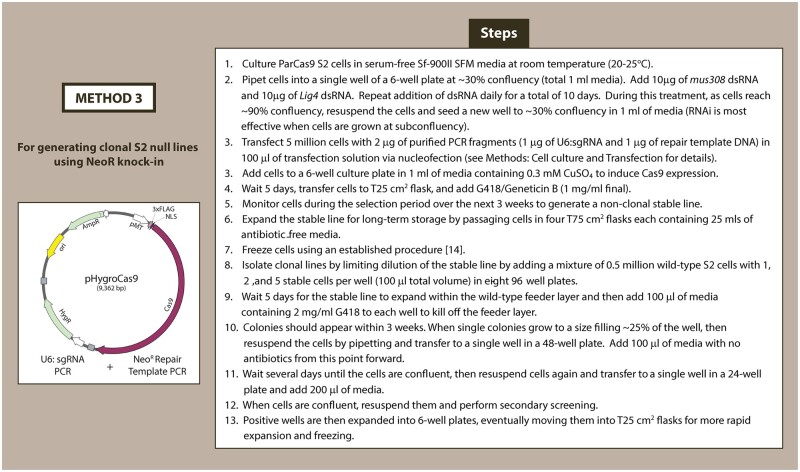
Outline of sequential steps for CRISPR–Cas9 knock-in to knock-out method via HDR in cultured S2 cells. This method describes a knock-in scheme of the neomycin-resistance gene to select for null mutants in the ParCas9 line.

Our third method uses knock-in of a neomycin-resistance gene to knock-out a gene-of-interest and generate clonal S2 null lines (Fig. 4). Similar to Method 2, we deplete Lig4 and mus308 by RNAi in ParCas9 cells prior to co-transfection with HR template and a sgRNA cassette. The HR template contains a Neo^R^ gene in-frame with the coding region of the gene-of-interest followed by a stop codon, similar in design to the insertion cassette used by Xia *et al*. [9]. After induction of Cas9 and followed by HDR, cells should express a neomycin-resistance gene from the locus of the CRISPR target gene. Our method uses inducible expression of Cas9 and transient expression of one sgRNA from a PCR product that is likely eliminated as cells divide. In contrast, the method by Xia *et al*. uses cells constitutively expressing Cas9 and four unique sgRNA [9], each of which has its own off-target potential. Additionally, our method uses far fewer cloning steps; we design primers with comparatively short homology arms (50 bp versus 1000 bp [9]), allowing us to synthesize HR templates by PCR and to target upstream sequences within the coding region of the gene without the risk of altering promoter regions to the HR template (our two proof-of-concepts both target sequence in the first exon). A disadvantage of both methods is the comparatively reduced likelihood of generating homozygous knock-in on all alleles (not directly measured in our study).

Neomycin-resistant, CRISPR-edited cells from Method 3 are plated at 1, 2, or 5 cells/well onto the feeder layer and screened for growth of single colonies (for more details, refer to Materials and methods section). We have found that selection with G418 is more effective at generating wells with single colonies compared to hygromycin, another advantage of Method 3 compared to Methods 1 or 2. These colonies can then be screened by PCR screening, Western blot, or phenotypic screening.

### Generating a Plk4-null line using knock-in to knock-out

We used our HDR-mediated Neo^R^ knock-in method (Fig. 4) to generate Polo-like kinase 4/Plk4 null S2 cell lines as a proof of concept. Plk4 is a conserved kinase in metazoans and the master regulator of centriole duplication [21]. Plk4’s catalytic activity promotes centriole duplication, and its overexpression is sufficient to induce the overproduction of centrioles (known as centriole amplification) [22–24]. Centrioles are the core components of centrosomes, the major microtubule-organizing center of the cell and centrosome copy number is controlled through tight Plk4 regulation. Specifically, Plk4 is regulated both transcriptionally and post-translationally to prevent its premature activation and, thus, suppress rampant centriole amplification [25–28]. Normally, Plk4 is autoinhibited when it is synthesized but then rapidly homodimerizes and *trans*-autophosphorylates its activation loop [29, 30]. However, Plk4 activation also stimulates its own destruction to prevent centriole amplification by *trans*-autophosphorylating a degron downstream of its kinase domain, resulting in recognition and ubiquitination by the Skp-Cullin-F-box E3 ubiquitin–ligase complex, SCF^Slimb^ [25, 26, 31, 32]. Upon entry to mitosis, Protein Phosphatase 2A (PP2A) counteracts Plk4 autophosphorylation of its degron [33], allowing Plk4 levels to rise and initiate the duplication process [17].

Due to the robust ability to promote its own autodestruction, Plk4 protein levels are undetectable throughout interphase, and therefore many studies of Plk4 regulation require overexpression of transgenes, leading to dramatically higher protein levels of Plk4 compared to the endogenous level [17]. However, as an indirect readout of endogenous Plk4 activity, one can measure centriole numbers simply by immunostaining for centriole proteins. Importantly, centrioles are non-essential organelles in S2 cells, and their loss does not trigger cell cycle arrest, unlike in human cells [34–37]. Moreover, in the absence of centrosomes, mitotic spindles can still form due to the acentrosomal spindle assembly pathway [38]. For example, centrioles fail to duplicate after RNAi-mediated depletion of Plk4 leading to a majority of cells lacking centrioles as remnant centrioles are diluted out of the proliferating cell culture [22]. In contrast, depletion of the F-box protein, Slimb, increases Plk4 levels (and, hence, its activity), leading to centriole amplification in S2 cells [17, 39].

**Figure 5.**
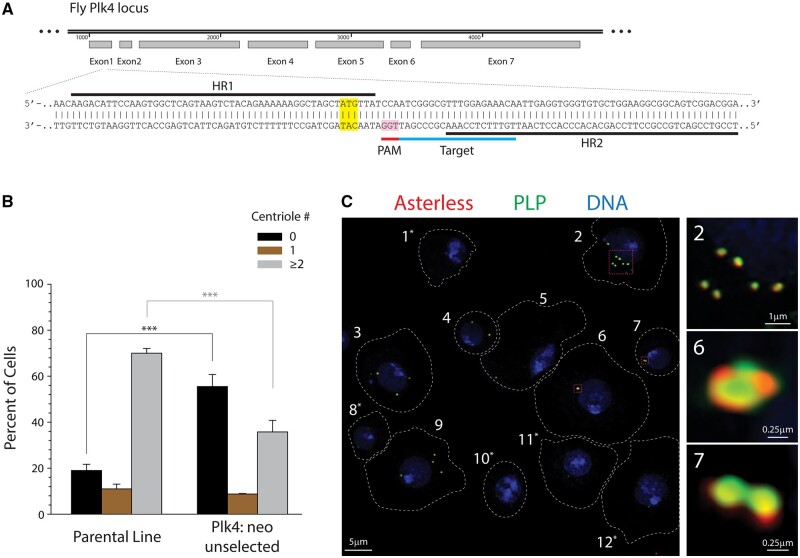
Application of Method 3: generating Plk4 null S2 cell lines. (A) Schematic of the Plk4 (SAK/CG7186) genomic locus on 3L (78D4-78D4) shows Exon 1 sequence targeted for knock-in of the neomycin-resistant (Neo^R^) gene. Although the Plk4 gene is on the anti-sense strand of Chromosome 3L, schematic has been oriented left-to-right for ease of presentation. Approximately 50 bp of HR flank the Neo^R^ repair template (data not shown). sgRNA (labeled Target) was designed toward bottom strand of the Plk4 locus using targetfinder.flycrispr.neuro.brown.edu to aid in target selection. The PAM and the Plk4 translation start site (ATG, highlight) are indicated. Successful knock-in should produce neomycin phosphotransferase II protein under control of the endogenous Plk4 promoter, using the first codons (ATG-TTA) of the Plk4 coding region. (B and C) A culture of heterogeneous stable Plk4 null S2 cells shows more cells containing zero centrioles compared to the parental (ParCas9) line. Completion of Step 5 (Fig. 4) produced a neomycin-resistant, stable S2 cell line (Plk4: neo). (B) The graph shows measurements of centrioles per cell. *n* = 100 cells in each of the three experiments. Bars represent the mean. Error bars, SEM. ***P < .001. (C) Interphase Plk4: neo S2 cells immunostained for centriole proteins Asterless/Asl and Plp. Dashed lines denote cell borders. Boxes show select cells at higher magnification (right). Cells numbered with asterisks lack centrioles. Cells 2, 3, and 9 display centriole amplification.

The Plk4 locus, SAK/CG7186 gene, is on the anti-sense strand of chromosome 3L (78D4–78D4) of the *Drosophila melanogaster* genome. We designed our sgRNA to target a PAM site within exon 1 downstream of the translation start site. Homology arms were constructed to insert the Neo^R^ gene in-frame with the Plk4 coding region, eliminate the gRNA binding site, and insert a frameshift downstream of the Neo^R^ gene (Fig. 5A). ParCas9 cells were depleted of Lig4 and mus308 for 10 days prior to transfection with PCR amplified sgRNA cassette and HR template. Upon inducing Cas9, we generated a stable line (Plk4: neo) with neomycin-resistance gene expression under the Plk4 promoter (Fig. 4, step 5).

We next measured centriole loss in our non-clonal Plk4: neo population to determine the efficiency of Plk4 knockout. Most S2 cells contain two centrioles, as observed by colocalization of the centriole markers Asterless (Asl) and Pericentrin-like Protein (Plp) (Fig. 5B). A subpopulation of cells in the ParCas9 line (∼20%) lack centrioles (Fig. 5B), typical of an untreated wild-type S2 cell culture [22]. However, after G418 selection, ∼55% of Plk4: neo cells lacked centrioles (Fig. 5B and C), suggesting that at least 35% of cells are Plk4 null.

### Generating clonal acentrosomal S2 cell lines

**Figure 6.**
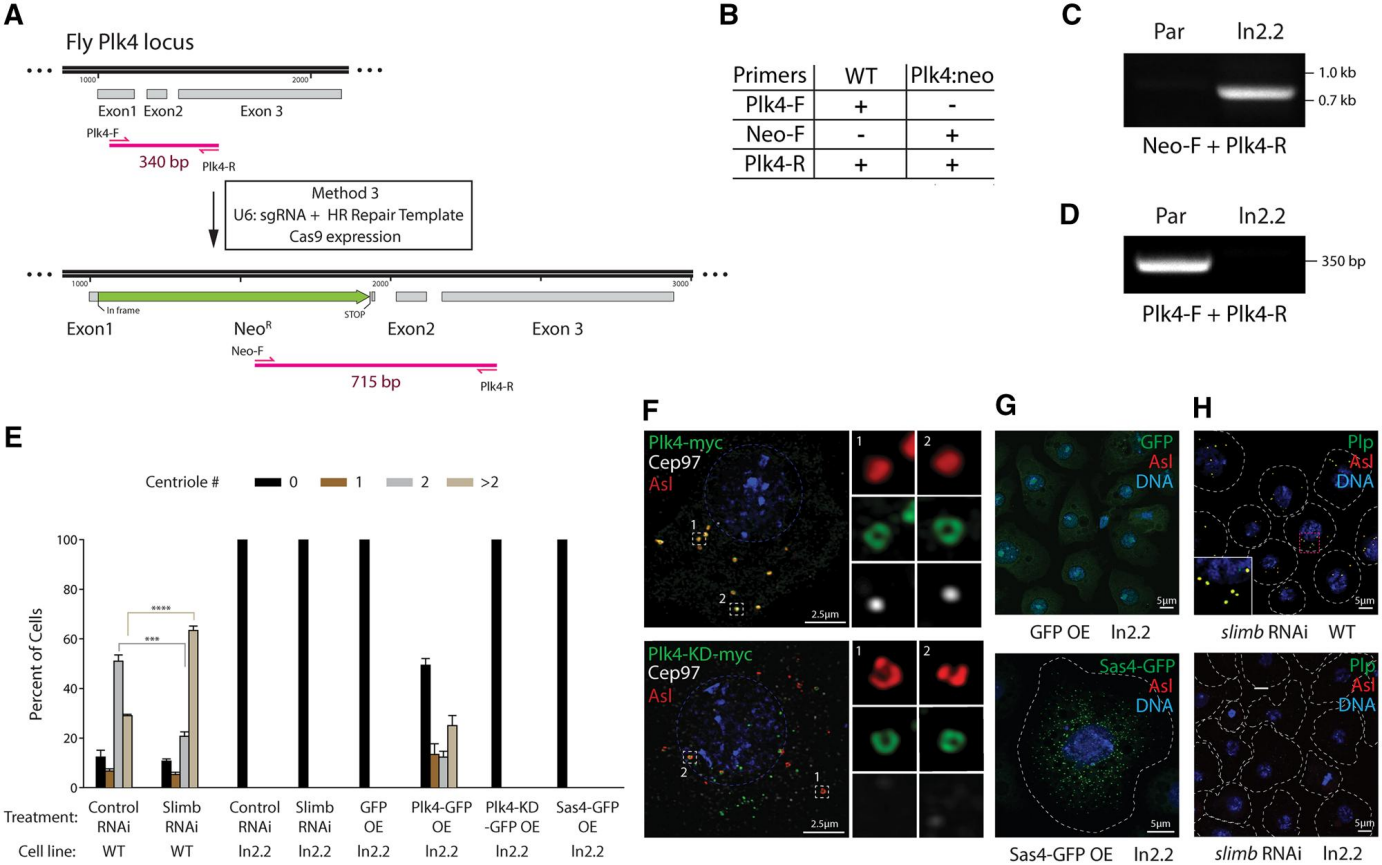
Completion of Method 3: characterization of a clonal Plk4 null S2 cell line. (A) (Upper panel) Map shows the Plk4 locus with exons 1–3. Plk4 gene-specific primers were designed at the site of insertion to PCR amplify a 340 bp fragment to identify wild-type, non-edited lines. (Lower panel) The map shows placement of CRISPR-edited knock-in of the neomycin-resistance gene (Neo^R^) within Exon 1 of the Plk4 gene. A stop codon at the end of Neo^R^ prevents translation of the Plk4 protein. Neo^R^ and Plk4 gene-specific primers were designed to PCR amplify a 715 bp fragment and identify a successful CRISPR knock-in. (B) Table lists primers that were used to identify the Neo^R^ knock-in and intact Plk4 gene shown in the schematics (A). (C) Gel shows PCR amplification of Neo^R^-Plk4 DNA fragment from the clonal Plk4 null 2.2 cell line but not from the parental ParCas9 line. (D) Gel shows PCR amplification of Plk4 DNA fragment from the parental ParCas9 line but not from the Plk4 null 2.2 cell line demonstrating that this line lacks intact copies of the endogenous Plk4 gene. (E) Graph shows measurements of centrioles per cell in the experiment described in D–F. *n* = 100 cells in each of the three experiments. Bars represent the mean. Error bars, SEM. ***P < .001; ****P < .0001. (F) Super-resolution microscopy of a Plk4 null cell expressing inducible wild-type Plk4-myc (upper panel) or Plk4-KD-myc (lower panel). Cells were transiently transfected with inducible wild-type Plk4-GFP, and after a 24-h recovery, were induced by the addition of 0.7 mM CuSO_4_ to culture media, and allowed to express for 3 days. Cells were then immunostained for Asl and Cep97 (which labels the distal tips of centrioles). Dashed lines denote the nuclei. Insets show select centrioles (boxes) at higher magnification. Note that overexpression of wild-type Plk4-myc rescues centriole loss in the Plk4 null line as indicated by the presence of numerous Cep97-positive centrioles. Interestingly, overexpression of KD Plk4-myc co-localizes with abnormally shaped Asl-labeled structures which are not centrioles because they lack Cep97. (G) Overexpression (OE) of inducible control GFP (upper panel) or centriole protein Sas4-GFP (lower panel) did not induce centriole amplification in the Plk4 null 2.2 line. Cells were transiently transfected with inducible wild-type Plk4-GFP, and after a 24 h recovery, were induced by the addition of 0.7 mM CuSO_4_ to culture media, and allowed to express for 3 days. Cells were then immunostained for Asl. Cell borders are marked with dashed lines. Note that overexpressed Sas4-GFP normally appears as numerous punctate aggregates in cells (as seen here); but the absence of co-localized centriolar Asl demonstrates that cells are acentriolar. (H) Whereas depletion of the F-box protein, Slimb, promotes centriole amplification in wild-type (WT) S2 cells (upper panel), Slimb depletion has no effect on centriole number in the Plk4 null 2.2 line (lower panel). S2 cell lines, ParCas9 and 2.2, were treated with dsRNA targeting Slimb for 7 days. RNAi-treated cells were then immunostained for centriole proteins Asterless/Asl and Plp. Cell borders are marked with dashed lines. The inset shows centriole amplification (box) at higher magnification.

Despite the high editing efficiency of our Plk4: neo line, our goal was to create a clonal Plk4 null line. Therefore, we performed single-cell selection using the Plk4: neo line. Cells 1, 2, and 5 were plated onto a feeder layer of 0.5 × 10^6^ wild-type S2 cells in each well of a 96-well plate (9 plates total). After 5 days, cells were treated with a final concentration of 1 mg/ml G418 to select for cells with the Neo^R^ insert. After 2–3 weeks, single colonies emerged in 4% of wells (26 total) and were expanded. Because Plk4 is required for centriole assembly, G418-resistant colonies were first phenotypically screened to identify lines that lacked centrioles by immunofluorescence (6 of 26 lines, 23% of lines; Supplementary Fig. S1B shows 3 lines that were further characterized). Next, to confirm knock-in of the Neo^R^ gene into the Plk4 locus within cell lines lacking centrioles, we designed primers spanning the Neo^R^ gene and downstream region of the endogenous Plk4 locus which will amplify a unique 715 bp region in Plk4: neo lines (Fig. 6A–C). To confirm editing of all copies of the Plk4 gene, we also designed PCR primers overlapping the endogenous Plk4 sequence that is disrupted by neomycin insertion, so that only unedited alleles contain the sequence necessary to prime PCR amplification (Fig. 6A, B, and D). We were unable to amplify PCR product from line 2.2, demonstrating that this line lacks wild-type copies of the Plk4 gene (Fig. 6D). Note: This does not confirm homozygous knock-in of Neo^R^ into all alleles; other copies may have been disrupted through residual end-joining pathways despite being depleted of Lig4 and mus308.

We next tested these lines using functional assays to confirm Plk4 knockout. First, we overexpressed a GFP-tagged Plk4 transgene in cells and found that Plk4 expression was sufficient to rescue centrosome loss in most transfected cells within these lines (Fig. 6E; Supplementary Fig. S1). However, expression of a kinase-dead (KD) Plk4 mutant failed to rescue the centriole loss phenotype and, instead, self-assembled into ring-like aggregates coated with Asl (Fig. 6E and F) [40, 41]. SoRa super-resolution microscopy revealed that Asl-containing Plk4-KD aggregates were not centrioles because they lacked the distal tip protein Cep97 that is found on bona fide centrioles and on centrioles generated by wild-type Plk4 overexpression [18, 24] (Fig. 6F). Overexpression of the conserved centriole surface protein, Sas4/CPAP, which normally promotes centrosome amplification in S2 cells containing Plk4 [42], also failed to rescue the centriole loss phenotype (Fig. 6E and G). Together, these suggest that Plk4 is the only missing component required for centriole assembly in these cells.

Although we were unable to determine whether all copies of the Plk4 gene contained a Neo^R^ gene knock-in, importantly, we were able to show that a Plk4: neo clone (line 2.2) lacked wild-type copies of the Plk4 gene (Fig. 6D). To further demonstrate that these clones were Plk4 null lines, we examined whether these cells produced any functional Plk4. Normally, Plk4 levels and activity are kept low through its ubiquitination by SCF^Slimb^ and subsequent degradation. However, Slimb depletion allows Plk4 levels to rise resulting in penetrant centrosome amplification (Fig. 6E and H), as previously described [17, 39]. Therefore, if any Plk4 was produced in the Plk4 null lines, then Slimb depletion should allow Plk4 levels to increase and promote centriole assembly over time. However, after 7 days of Slimb RNAi, centrioles did not form in the Plk4 null lines (Fig. 6E and H), strongly suggesting that these are full Plk4 knock-outs.

**Figure 7.**
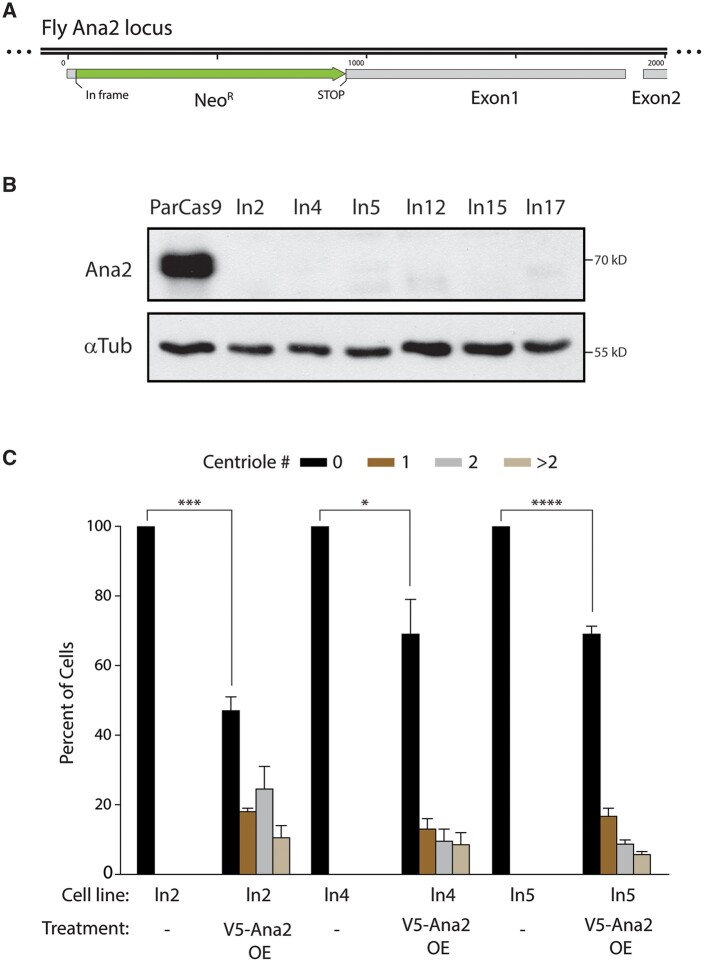
Application of Method 3: generating Ana2 null S2 cell lines. (A) Map of the Ana2 (CG8262) genomic locus on 2R (44F3–44F3) shows placement of CRISPR-edited knock-in of the neomycin-resistance gene (Neo^R^) within Exon 1 of the Ana2 gene. Although the Ana2 gene is on the anti-sense strand of Chromosome 2R, schematic has been oriented left-to-right for ease of presentation. A stop codon at the end of Neo^R^ prevents translation of the Ana2 protein immediately followed by frameshift of the Ana2 coding frame. Successful knock-in should produce neomycin phosphotransferase II protein under control of the endogenous Ana2 promoter, using the first 12 codons (ATG-TTT-GTT-CCC-GAA-ACG-GAG-GAT-ATG-CTG-CCC-AGA) of Ana2 coding region. (B) A long exposure of an anti-Ana2 immunoblot of six different clonal Ana2 null S2 cell lines demonstrates complete elimination of endogenous Ana2 protein. Tubulin, loading control. (C) Overexpression (OE) of V5-tagged Ana2 restores centriole assembly in clonal populations of Ana2 CRISPR null S2 cells. Cells were transfected with inducible wild-type V5-Ana2, recovered for 24 h, and then induced by the addition of 0.7 mM CuSO_4_ to culture media, and allowed to express for 3 days. Cells were then immunostained for Asterless/Asl and centrioles per cell were counted. The graph shows measurements of centrioles per cell in three different clonal Ana2 CRISPR null cell lines. *n* = 100 cells in each of the three experiments. Error bars, SEM. **P < .01.

Nonetheless, since endogenous Plk4 protein is undetectable in S2 cell lysates, we continued our proof of concept by knocking-out a different centriole gene whose protein levels are relatively abundant and easily observable by immunoblot. *Drosophila* Anastral Spindle 2/Ana2 (STIL in humans) is a structural centriole component essential for centriole duplication [16, 43–48]. Using the same knock-in to knock-out method, we designed our sgRNA to target a PAM site within exon 1 of the Ana2 gene on the anti-sense strand of chromosome 2R (44F3–44F3). As before, the HR template was designed to insert the Neo^R^ gene in frame with the Ana2 coding region, resulting in the retention of the first 12 codons of Ana2 (Fig. 7A). The Neo^R^ contained its own stop codon and introduced a frameshift immediately downstream. After inducing Cas9 expression and establishing a stable, neomycin-resistant Ana2 null line, we found that 71% of these cells contained zero centrioles (compared to ∼20% in the ParCas9 lines). The Ana2: neo population was then clonally expanded and 14% of wells grew single colonies (109 of 768 wells). Since Ana2 is required for centriole duplication, we screened 18 clonal lines for centriole loss by immunostaining for Asl and Plp. Nearly half of the lines (8 of 18) lacked centrioles, suggesting a full knockout of the 4 copies of the Ana2 gene in the S2 cell genome [15]. Next, of the eight positives, we randomly selected six clonal lines, prepared cell lysates, and immunoblotted for Ana2. Notably, we did not detect endogenous Ana2 in any of these cells, confirming the Ana2 null status of the clonal lines (Fig. 7B). Lastly, overexpression of transgenic V5-tagged Ana2 significantly rescued centrosome loss in all three of the Ana2 null lines that we examined (Fig. 7C). Taken together, our CRISPR knock-in to knock-out method efficiently depleted Ana2 in S2 cells, as 44% of clonal lines were acentrosomal.

## Discussion

Here we provide a straightforward, easy-to-replicate system of CRISPR editing in *Drosophila* S2 cells. Notably, we designed an alternative approach for generating clonal null lines (Figs 4, 5A, and 6D). We described this method as a “knock-in to knock-out,” which entails the insertion of an antibiotic resistance gene (with a 3′ stop codon) just downstream of the translation start site of the target gene. This insertion prevents translation of the targeted gene, and additionally facilitates the selection of cells containing in-frame insertions; although the identification of complete knockout lines requires a secondary screening step. This method will be a great tool added to the current collection of CRISPR methods for *Drosophila* culture cells, in addition to our knock-in method of tags and fluorescent reporters described in Fig. 3.

There are important considerations and limitations of using CRISPR editing methods in fly cell lines. First, Cas9 efficiency and off-target effects must be a concern. Off-target rates were not measured in our study but, to minimize off-target effects, we used *in silico* prediction tools to select gRNAs with the fewest off-target effects. Additionally, we used only one sgRNA to generate our lines, while other studies used up to four sgRNA, compounding their off-target risk [9, 49]. Off-target effects can also increase with long-term Cas9 expression [50, 51], therefore we designed this system to use transient expression of a sgRNA and inducible expression of Cas9 to limit the duration of genome editing. We also used 20 nt gRNAs for these methods; however, previous studies have shown that gRNAs smaller than 20 nt improve on-target efficiency [52]. With the methodology outlined in this study, different length gRNAs can be easily tested and explored. Finally, our study used the human codon-optimized form of spCas9, further improvements could be made by using high-fidelity Cas9 variants that perform better in *Drosophila* cells (reviewed in [49]).

Rational design of the HR template is a key component of this method. The neomycin-resistance gene lacks its own promoter or start codon, therefore antibiotic treatment selects for cells with at least one allelic knock-in, meaning cells have confidently been transfected with sgRNA cassette and HR template and that these cells will repair DSB using HR. However, like the insertion cassette described by Xia *et al*. [9], the reliance of Method 3 on the target gene’s endogenous promoter means that genes expressed in low levels may not be feasible candidates for editing with Method 3. Additionally, the short homology arms allow the Neo^R^ gene to be inserted early in the gene coding region, thus reducing the risk that translated fragments of the protein are functional. Finally, introducing a frameshift after the Neo^R^ gene stop codon should prevent the expression of the endogenous gene due to aberrant ribosome readthrough [53].

Isolating clonal *Drosophila* lines (such as S2) is a challenging but rewarding task. Combining CRISPR editing with single-cell cloning allows researchers the ability to work with a homogenous and stable cell model. The clonal expansion of single cultured fly cells in conditioned media and serum-containing media has been described with limiting dilutions and after FACS sorting [8, 9, 12]. However, in our hands, these methods do not work in establishing clonal lines as individual cells do not grow, even in the small wells of 384-well plates. Instead, we use limiting dilution of antibiotic-resistant cells onto live, wild-type feeder layers that are then eliminated by antibiotic selection. We find that this consistently results in wells containing single colonies of cells. While a limitation of this study is the assumption that a single colony arose from a single cell, the likelihood of a colony containing multiple clones that are all knock-outs is theoretically low, given an approximate 23%–44% knock-in to knock-out efficiency.

As proof of concept, we used the knock-in to knock-out method in S2 cells and targeted the Plk4 and Ana2 genes, generating the first acentrosomal S2 lines to date. Because S2 cells can divide without their centrioles, this cell line is ideal for studying Plk4 and Ana2 regulation and the mechanics of centriole assembly. Notably, secondary screening is required to confirm the loss of all endogenous gene copies and, using this method, we observed a 23% efficiency of Plk4 knockout and a 44% efficiency of Ana2 knockout; centrioles were absent in these lines and only expression of a Plk4 or Ana2 transgene could rescue centriole loss (Figs 6E and 7C; Supplementary Fig. S1B). A disadvantage to our method is that is a lengthy process, taking 2–3 months to establish a CRISPR clonal line, but this timeline is similar to other published methods [9, 54].

The three methods of CRISPR/Cas9 genome editing we describe here add to the already well-described methods in *Drosophila* cells. This system can theoretically be used toward any non-essential *Drosophila* gene in any transfectable fly cell line. Our CRISPR system is easy to establish, cost-effective and efficient. The use of selection markers overcomes dose-dependent issues arising from the aneuploid *Drosophila* genome and makes the generation of clonal lines simple.

## Supplementary Material

bpae059_Supplementary_Data

## Data Availability

The data underlying this article are available in the article and in its online supplementary material. Reagents will be shared on reasonable request to the corresponding author.
