# Supplementary material for: Generating CRISPR-edited clonal lines of cultured Drosophila S2 cells
Source: Biol Methods Protoc. 2024 Aug 17;9(1):bpae059. doi: 10.1093/biomethods/bpae059 (PMC11357795; doi:10.1093/biomethods/bpae059)
Supplement: bpae059_Supplementary_Data [file bpae059_supplementary_data.pdf]

Figure S1

A

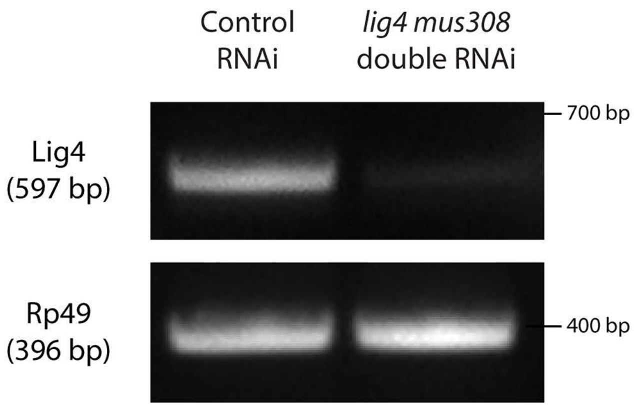
Control RNAi
lig4 mus308 double RNAi
Lig4 (597 bp)
Rp49 (396 bp)
700 bp
400 bp

B

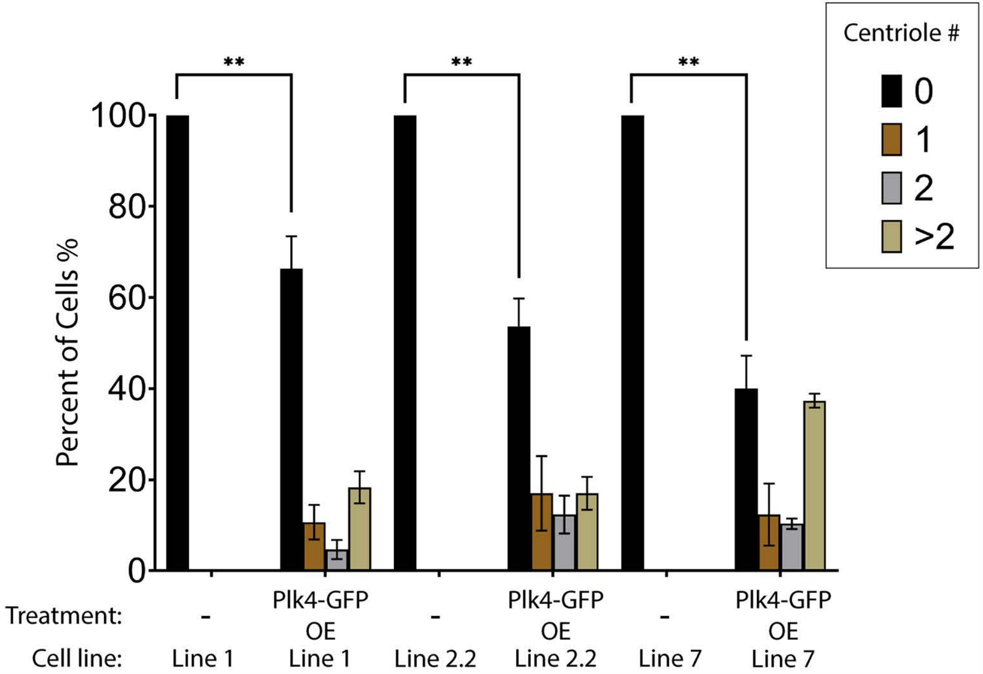
Centriole #
0
1
2
>2
**
**
**
Percent of Cells %
100
80
60
40
20
0
Treatment:
-
Plk4-GFP OE
-
Plk4-GFP OE
-
Plk4-GFP OE
Cell line:
Line 1
Line 1
Line 2.2
Line 2.2
Line 7
Line 7

Supplementary Table S1: Reagents and resources

REAGENT or RESOURCE	SOURCE	IDENTIFIER
Antibodies		
Rat polyclonal anti-Asl antibodies	Rogers Lab	Rogers Lab
Rabbit polyclonal anti-Plp antibodies	Rogers Lab	Rogers Lab
Chicken polyclonal anti-Cep97 antibodies	Rogers Lab	Rogers Lab
AlexaFluor 488 donkey anti-rabbit IgG	Thermo Fisher Scientific	Cat# A21206
Rhodamine Red-X AffiniPure goat anti-rat IgG	Jackson Immunoresearch	Cat# 112-295-143
Alexa Fluor Plus 647 goat anti-chicken	Thermo Fisher Scientific	Cat# A32933
Bacterial strains		
5-alpha competent *E.coli*	New England Biolabs	Cat# C29871
GC5 Competent Bacteria	Genesee Scientific	Cat# 42-650
Chemicals, peptides, enzymes, and recombinant proteins		
Neomycin G418	Thermo Fisher Scientific	Cat# J62671.03
Hygromycin	GoldBiotech	Cat# H-270-1
EcoRI	NEB	Cat# R0101S
HindIII	NEB	Cat# R0104S
KpnI-HF	NEB	Cat# R3142S
Normal Goat Serum	Sigma Aldrich	Cat# G9023
Triton X-100	Thermo Fisher Scientific	Cat# BP151-500
Paraformaldehyde 16% Solution	Electron Microscopy Sciences	Cat#15710
IRDye® 800CW Streptavidin	Li-Cor	Cat# C91204-06
Vectashield	Vector Laboratories	Cat# H-1000

Phusion High-Fidelity DNA Polymerase	Thermo Fisher Scientific	Cat# F530L
Hoechst 33342	Life Technologies	Cat# H3570
Sf-900 II SFM	Gibco	Cat# 10902-088
Penicillin Streptomycin Solution	Corning	Cat# 30-002-CI
RNase-Free DNase	Qiagen	Cat# P79254
Phenylmethanesulfonyl fluoride	Sigma-Aldrich	Cat# P7626
Critical commercial assays		
T7 RiboMAX™ Large Scale RNA Production System	Promega	Cat# P1300
SuperScript III One-Step system	Invitrogen	Cat# 12574-018
Gateway LR Clonase	Thermo Fisher Scientific	Cat# 11791043
Plasmids		
pAc-sgRNA-Cas9	Addgene	Cat# 49330
Experimental models: Cell lines		
D. melanogaster: Cell line S2	Thermo Fisher Scientific	Cat# R69007
pMT:3xFLAG-Cas9	Rogers Lab	Rogers Lab
Oligonucleotides		
Slimb RNAi F:<br>GGCCGCCACATGCTGCG	Rogers Lab	Rogers Lab
Slimb RNAi R:<br>CGGTCTTGTTCTCATTGGG	Rogers Lab	Rogers Lab
U6 promoter sequence:<br>GGTATGTTTTCCTCAATACTTCG	IDTDNA	N/A
SV40 polyA tail:<br>GTTTTAGAGCTAGAAATAGC	IDTDNA	N/A

Cas9-1:<br><br>CGGGGTACCATGGACTATAAGGACCACGACGG	IDTDNA	N/A
Cas9-2:<br><br>CCGGAATTCTTAGTCGCCTCCCAGCTGAGACAGG	IDTDNA	N/A
Cas9 Screening Forward:<br><br>AGCCTGGGCCTGACCCC	IDTDNA	N/A
Cas9 Screening Reverse:<br><br>TCTTAGGGTCCCAGTCC	IDTDNA	N/A
gRNA cassette cloning primer 1:<br><br>TCCCGGAGACGGTCACAGCATCTGTTCGACTTGCA GCCTGAAATACG	IDTDNA	N/A
gRNA cassette cloning primer 2:<br><br>GCTTGTCTGCTCCCGGCATCCGCGGCGTATCACGA GGCCCTTTCG	IDTDNA	N/A
gRNA sequencing primer:<br><br>GCAGAGGGTTCTTAAGACC	IDTDNA	N/A
Plk4 gRNA F:<br>GGTATGTTTTCCTCAATACTTCGGGTCTTCGTCCGA TTGTGACGTTTTAGAGCTAGAAATAGC	IDTDNA	N/A
Plk4 gRNA R:<br>GCTATTTCTAGCTCTAAAACGTCACAATCGGACGAA GACCCGAAGTATTGAGGAAAACATACC	IDTDNA	N/A
Plk4-neo HR- F:<br>AAGACATTCCAAGTGGCTCAGTAAGTCTACAGAAAA AAGGCTAGCTATGTTAATTGAACAAGATGGATTGC	IDTDNA	N/A
Plk4-neo HR- R:<br>TCCGTCCGACTGCCGCCTTCCAGCACACCCACCTC AATTGTTTCTCCAAATCAGAAGAACTCGTCAAGAAG GCG	IDTDNA	N/A
U6-F:<br><br>ATCTGTTCGACTTGCAGCC	IDTDNA	N/A

polyA-R: GGCGTATCACGAGGCCC	IDTDNA	N/A
Plk4-screen-F: TATGTTATCCAATCGGGC	IDTDNA	N/A
lig4-F: CGGCTCATCCTTCAACAGCCACGC	IDTDNA	N/A
lig4-R: GGAAGTAGGATGCCTTCGCGATGGC	IDTDNA	N/A
Rp49 – For: ATCCGCCCAGCATACAGG	Rogers Lab	Rogers Lab
Rp49 – Rev: CTCGTTCTCTTGAGAACGCAG	Rogers Lab	Rogers Lab
Recombinant DNA		
pMT/V5-HisC	Life Technologies	Cat#K4120-01
pMT/V5 His C GFP	Rogers Lab	Rogers Lab
pMT/V5 His C Plk4-GFP	Rogers Lab	Rogers Lab
pMT/V5 His C Plk4-Kinase Dead-GFP	Rogers Lab	Rogers Lab
pMT/V5 His C Plk4-myc	Rogers Lab	Rogers Lab
pMT/V5 His C Sas4-GFP	Rogers Lab	Rogers Lab
pHygroCas9	Rogers Lab	Rogers Lab
pHygroCas9-U6gRNA	Rogers Lab	Rogers Lab
pAc-sgRNA-Cas9	Addgene	Cat# 49330
Software and algorithms		
Prism 7	GraphPad	www.graphpad.com/scientificsoftware/prism/
CRISPR Optimal Target Finder		www.targetfinder.flycrispr.neuro.brown.edu

Elements	Nikon	
FIJI / ImageJ	NIH	http://fiji.sc/
Zen Black	Zeiss	
Adobe Illustrator	Adobe	
Adobe Photoshop	Adobe	
Softworx	Cytiva	
Other		

Supplementary Table S2

Plasmid Sequences

**pHygroCas9** - 3xFLAG-NLS-Cas9, gray highlight; Hygromycin resistance gene, pink highlight.
TCGCGCGTTTCGGTGATGACGGTGAAAACCTCTGACACATGCAGCTCCCGGAGACGGTCACAGCTTGTCTGTAAGC<br>GGATGCCGGGAGCAGACAAGCCCGTCAGGGCGCGTCAGCGGGTGTTGGCGGGTGTCGGGGCTGGCTTAACTATGCG<br>GCATCAGAGCAGATTGTACTGAGAGTGCACCATATGCGGTGTGAAATACCGCACAGATGCGTAAGGAGAAAATACC<br>GCATCAGGCGCCATTCGCCATTCAGGCTGCGCAACTGTTGGGAAGGGCGATCGGTGCGGGCCTCTTCGCTATTACG<br>CCAGCTGGCGAAAGGGGGATGTGCTGCAAGGCGATTAAGTTGGGTAACGCCAGGGTTTTCCCAGTCACGACGTTGT<br>AAAACGACGGCCAGTGCCAAGCTTCGTTGCAGGACAGGATGTGGTGCCCGATGTGACTAGCTCTTTGCTGCAGGCC<br>GTCCTATCCTCTGGTTCCGATAAGAGACCCAGAACTCCGGCCCCCCACCGCCCACCGCCACCCCCATACATATGTG<br>GTACGCAAGTAAGAGTGCCTGCGCATGCCCCATGTGCCCCACCAAGAGTTTTGCATCCCATACAAGTCGCCAAAGT<br>GGAGAACCGAACCAATTCTTCGCGGGCAGAACAAAACCTTCTGCACACGTCTCCACTCGAATTTGGAGCCGGCCGG<br>CGTGTGCAAAAGAGGTGAATCGAACGAAAGACCCGTGTGTAAAGCCGCGTTTCCAAAATGTATAAAACCGAGAGCA<br>TCTGGCCAATGTGCATCAGTTGTGGTCAGCAGCAAAATCAAGTGAATCATCTCAGTGCAACTAAacaccATGGACT<br>ATAAGGACCACGACGGAGACTACAAGGATCATGATATTGATTACAAAGACGATGACGATAAGGCCCCAAAGAAGAA<br>GCGGAAGGTCGGTATCCACGGAGTCCCAGCAGCCGACAAGAAGTACAGCATCGGCCTGGACATCGGCACCAACTCT<br>GTGGGCTGGGCCGTGATCACCGACGAGTACAAGGTGCCCAGCAAGAAATTCAAGGTGCTGGGCAACACCGACCGGC<br>ACAGCATCAAGAAGAACCTGATCGGAGCCCTGCTGTTCGACAGCGGCGAAACAGCCGAGGCCACCCGGCTGAAGAG<br>AACCGCCAGAAGAAGATACACCAGACGGAAGAACCGGATCTGCTATCTGCAAGAGATCTTCAGCAACGAGATGGCC<br>AAGGTGGACGACAGCTTCTTCCACAGACTGGAAGAGTCCTTCCTGGTGGAAGAGGATAAGAAGCACGAGCGGCACC<br>CCATCTTCGGCAACATCGTGGACGAGGTGGCCTACCACGAGAAGTACCCCACCATCTACCACCTGAGAAAGAAACT<br>GGTGGACAGCACCGACAAGGCCGACCTGCGGCTGATCTATCTGGCCCTGGCCCACATGATCAAGTTCCGGGGCCAC<br>TTCCTGATCGAGGGCGACCTGAACCCCGACAACAGCGACGTGGACAAGCTGTTCATCCAGCTGGTGCAGACCTACA<br>ACCAGCTGTTCGAGGAAAACCCCATCAACGCCAGCGGCGTGGACGCCAAGGCCATCCTGTCTGCCAGACTGAGCAA<br>GAGCAGACGGCTGGAAAATCTGATCGCCCAGCTGCCCGGCGAGAAGAAGAATGGCCTGTTCGGAAACCTGATTGCC<br>CTGAGCCTGGGCCTGACCCCCAACTTCAAGAGCAACTTCGACCTGGCCGAGGATGCCAAACTGCAGCTGAGCAAGG<br>ACACCTACGACGACGACCTGGACAACCTGCTGGCCCAGATCGGCGACCAGTACGCCGACCTGTTTCTGGCCGCCAA<br>GAACCTGTCCGACGCCATCCTGCTGAGCGACATCCTGAGAGTGAACACCGAGATCACCAAGGCCCCCCTGAGCGCC<br>TCTATGATCAAGAGATACGACGAGCACCACCAGGACCTGACCCTGCTGAAAGCTCTCGTGCGGCAGCAGCTGCCTG<br>AGAAGTACAAAGAGATTTTCTTCGACCAGAGCAAGAACGGCTACGCCGGCTACATTGACGGCGGAGCCAGCCAGGA<br>AGAGTTCTACAAGTTCATCAAGCCCATCCTGGAAAAGATGGACGGCACCGAGGAACTGCTCGTGAAGCTGAACAGA<br>GAGGACCTGCTGCGGAAGCAGCGGACCTTCGACAACGGCAGCATCCCCCACCAGATCCACCTGGGAGAGCTGCACG<br>CCATTCTGCGGCGGCAGGAAGATTTTTACCCATTCCTGAAGGACAACCGGGAAAAGATCGAGAAGATCCTGACCTT<br>CCGCATCCCCTACTACGTGGGCCCTCTGGCCAGGGGAAACAGCAGATTCGCCTGGATGACCAGAAAGAGCGAGGAA<br>ACCATCACCCCCTGGAACTTCGAGGAAGTGGTGGACAAGGGCGCTTCCGCCCAGAGCTTCATCGAGCGGATGACCA<br>ACTTCGATAAGAACCTGCCCAACGAGAAGGTGCTGCCCAAGCACAGCCTGCTGTACGAGTACTTCACCGTGTATAA<br>CGAGCTGACCAAAGTGAAATACGTGACCGAGGGAATGAGAAAGCCCGCCTTCCTGAGCGGCGAGCAGAAAAAGGCC<br>ATCGTGGACCTGCTGTTCAAGACCAACCGGAAAGTGACCGTGAAGCAGCTGAAAGAGGACTACTTCAAGAAAATCG<br>AGTGCTTCGACTCCGTGGAAATCTCCGGCGTGGAAGATCGGTTCAACGCCTCCCTGGGCACATACCACGATCTGCT<br>GAAAATTATCAAGGACAAGGACTTCCTGGACAATGAGGAAAACGAGGACATTCTGGAAGATATCGTGCTGACCCTG<br>ACACTGTTTGAGGACAGAGAGATGATCGAGGAACGGCTGAAAACCTATGCCCACCTGTTCGACGACAAAGTGATGA<br>AGCAGCTGAAGCGGCGGAGATACACCGGCTGGGGCAGGCTGAGCCGGAAGCTGATCAACGGCATCCGGGACAAGCA<br>GTCCGGCAAGACAATCCTGGATTTCCTGAAGTCCGACGGCTTCGCCAACAGAAACTTCATGCAGCTGATCCACGAC<br>GACAGCCTGACCTTTAAAGAGGACATCCAGAAAGCCCAGGTGTCCGGCCAGGGCGATAGCCTGCACGAGCACATTG<br>CCAATCTGGCCGGCAGCCCCGCCATTAAGAAGGGCATCCTGCAGACAGTGAAGGTGGTGGACGAGCTCGTGAAAGT<br>GATGGGCCGGCACAAGCCCGAGAACATCGTGATCGAAATGGCCAGAGAGAACCAGACCACCCAGAAGGGACAGAAG<br>AACAGCCGCGAGAGAATGAAGCGGATCGAAGAGGGCATCAAAGAGCTGGGCAGCCAGATCCTGAAAGAACACCCCG<br>TGGAAAACACCCAGCTGCAGAACGAGAAGCTGTACCTGTACTACCTGCAGAATGGGCGGGATATGTACGTGGACCA<br>GGAACTGGACATCAACCGGCTGTCCGACTACGATGTGGACCATATCGTGCCTCAGAGCTTTCTGAAGGACGACTCC<br>ATCGACAACAAGGTGCTGACCAGAAGCGACAAGAACCGGGGCAAGAGCGACAACGTGCCCTCCGAAGAGGTCGTGA<br>AGAAGATGAAGAACTACTGGCGGCAGCTGCTGAACGCCAAGCTGATTACCCAGAGAAAGTTCGACAATCTGACCAA

GGCCGAGAGAGGCGGCCTGAGCGAACTGGATAAGGCCGGCTTCATCAAGAGACAGCTGGTGGAAACCCGGCAGATC
ACAAAGCACGTGGCACAGATCCTGGACTCCCGGATGAACACTAAGTACGACGAGAATGACAAGCTGATCCGGGAAG
TGAAAGTGATCACCCTGAAGTCCAAGCTGGTGTCCGATTTCCGGAAGGATTTCCAGTTTTACAAAGTGCGCGAGAT
CAACAACTACCACCACGCCCACGACGCCTACCTGAACGCCGTCGTGGGAACCGCCCTGATCAAAAAGTACCCTAAG
CTGGAAAGCGAGTTCGTGTACGGCGACTACAAGGTGTACGACGTGCGGAAGATGATCGCCAAGAGCGAGCAGGAAA
TCGGCAAGGCTACCGCCAAGTACTTCTTCTACAGCAACATCATGAACTTTTTCAAGACCGAGATTACCCTGGCCAA
CGGCGAGATCCGGAAGCGGCCTCTGATCGAGACAAACGGCGAAACCGGGGAGATCGTGTGGGATAAGGGCCGGGAT
TTTGCCACCGTGCGGAAAGTGCTGAGCATGCCCCAAGTGAATATCGTGAAAAAGACCGAGGTGCAGACAGGCGGCT
TCAGCAAAGAGTCTATCCTGCCCAAGAGGAACAGCGATAAGCTGATCGCCAGAAAGAAGGACTGGGACCCTAAGAA
GTACGGCGGCTTCGACAGCCCCACCGTGGCCTATTCTGTGCTGGTGGTGGCCAAAGTGGAAAAGGGCAAGTCCAAG
AAACTGAAGAGTGTGAAAGAGCTGCTGGGGATCACCATCATGGAAAGAAGCAGCTTCGAGAAGAATCCCATCGACT
TTCTGGAAGCCAAGGGCTACAAAGAAGTGAAAAAGGACCTGATCATCAAGCTGCCTAAGTACTCCCTGTTCGAGCT
GGAAAACGGCCGGAAGAGAATGCTGGCCTCTGCCGGCGAACTGCAGAAGGGAAACGAACTGGCCCTGCCCTCCAAA
TATGTGAACTTCCTGTACCTGGCCAGCCACTATGAGAAGCTGAAGGGCTCCCCCGAGGATAATGAGCAGAAACAGC
TGTTTGTGGAACAGCACAAGCACTACCTGGACGAGATCATCGAGCAGATCAGCGAGTTCTCCAAGAGAGTGATCCT
GGCCGACGCTAATCTGGACAAAGTGCTGTCCGCCTACAACAAGCACCGGGATAAGCCCATCAGAGAGCAGGCCGAG
AATATCATCCACCTGTTTACCCTGACCAATCTGGGAGCCCCTGCCGCCTTCAAGTACTTTGACACCACCATCGACC
GGAAGAGGTACACCAGCACCAAAGAGGTGCTGGACGCCACCCTGATCCACCAGAGCATCACCGGCCTGTACGAGAC
ACGGATCGACCTGTCTCAGCTGGGAGGCGACtgaGTTTAAACCCGCTGATCAGCCTCGACTGTGCCTTCTAAGATC
CAGACATGATAAGATACATTGATGAGTTTGGACAAACCACAACTAGAATGCAGTGAAAAAAATGCTTTATTTGTGA
AATTTGTGATGCTATTGCTTTATTTGTAACCATTATAAGCTGCAATAAACAAGTTAACAACAACAAAGCTTGCAT
GCCTGCAGGTCGACTCTAGAGGATCCGGTGCGTGGTGGTTTCATGCTTCTGGGAACGGCAAATGGGTTTAGGATTG
GGAACCCCTCATCATCTGTTGGAATATACTATTCAACCTACAAAAATAACGTTAAACAACACTACTTTATATTTGA
TATGAATGGCCACACCTTTTATGCCATAAAACATATTGTAAGAGAATACCACTCTTTTTATTCCTTCTTTCCTTCT
TGTACGTTTTTTGCTGTGAGTAGGTCGTGGTGCTGGTGTTGCAGTTGAAATAACTTAAAATATAAATCATAAAACT
CAAACATAAACTTGACTATTTATTTATTTATTAAGAAAGGAAATATAAATTATAAATTACAACAGGTTATGGGGAT
CCCCAGCTTGGCCACCATGGGAAAGCCTGAGCGTACCGCGACGTCTGTCGAGAAGTTTCTGATCGAAAAGTTCGAC
AGCGTCTCCGACCTGATGCAGCTCTCGGAGGGCGAAGAATCTCGTGCTTTCAGCTTCGATGTAGGAGGGCGTGGAT
ATGTCCTGCGGGTAAATAGCTGCGGCGATGGTTTCTACAAAGATCGTTATGTTTATCGGCACTTTGCATCGGCCGC
GCTCCCGATTCCGGAAGTGCTTGACATTGGGGAATTCAGCGAGAGCCTGACCTATTGCATCTCCCGCCGTGCACAG
GGTGTCACGTTGCAAGACCTGCCTGAAACCGAACTGCCCGCTGTTCTGCAGCCGGTCGCGGAGGCCATGGATGCGA
TCGCTGCGGCCGATCTTAGCCAGACGAGCGGGTTCGGCCCATTCGGACCGCAAGGAATCGGTCAATACACTACATG
GCGTGATTTCATATGCGCGATTGCTGATCCCCATGTGTATCACTGGCAAACTGTGATGGACGACACCGTCAGTGCG
TCCGTCGCGCAGGCTCTCGATGAGCTGATGCTTTGGGCCGAGGACTGCCCCGAAGTCCGGCACCTCGTGCACGCGG
ATTTCGGCTCCAACAATGTCCTGACGGACAATGGCCGCATAACAGCGGTCATTGACTGGAGCGAGGCGATGTTCGG
GGATTCCCAATACGAGGTCGCCAACATCTTCTTCTGGAGGCCGTGGTTGGCTTGTATGGAGCAGCAGACGCGCTAC
TTCGAGCGGAGGCATCCGGAGCTTGCAGGATCGCCGCGGCTCCGGGCGTATATGCTCCGCATTGGTCTTGACCAAC
TCTATCAGAGCTTGGTTGACGGCAATTTCGATGATGCAGCTTGGGCGCAGGGTCGATGCGACGCAATCGTCCGATC
CGGAGCCGGGACTGTCGGGCGTACACAAATCGCCCGCAGAAGCGCGGCCGTCTGGACCGATGGCTGTGTAGAAGTA
CTCGCCGATAGTGGAAACCGACGCCCCAGCACTCGTCCGAGGGCAAAGGAATAGAGTAGATGCCGACCGAACAAGA
GCTGATTTCGAGAACGCCTCAGCCAGCAACTCGCGCGAGCCTAGCAAGGCAAATGCGAGAGAACGGCCTTACGCTT
GGTGGCACAGTTCTCGTCCACAGTTCGCTAAGCTCGCTCGGCTGGGTCGCGGGAGGGCCGGTCGCAGTGATTCAGG
CCCTTCTGGATTGTGTTGGTCCCCAGGGCACGATTGTCATGCCCACGCACTCGGGTGATCTGACTGATCCCGCAGA
TTGGAGATCGCCGCCCGTGCCTGCCGATTGGGTGCAGATCAGCCTCGAGGCCAGCTAGCTTGAACTTGTTTATTGC
AGCTTATAATGGTTACAAATAAAGCAATAGCATCACAAATTTCACAAATAAAGCATTTTTTTCACTGCATTCTAGT
TGTGGTTTGTCCAAACTCATCAATGTATCTTATCATGTCTGGATCGGGTACCGAGCTCGAATTCGTAATCATGTCA
TAGCTGTTTCCTGTGTGAAATTGTTATCCGCTCACAATTCCACACAACATACGAGCCGGAAGCATAAAGTGTAAAG
CCTGGGGTGCCTAATGAGTGAGCTAACTCACATTAATTGCGTTGCGCTCACTGCCCGCTTTCCAGTCGGGAAACCT
GTCGTGCCAGCTGCATTAATGAATCGGCCAACGCGCGGGGAGAGGCGGTTTGCGTATTGGGCGCTCTTCCGCTTCC
TCGCTCACTGACTCGCTGCGCTCGGTCGTTCGGCTGCGGCGAGCGGTATCAGCTCACTCAAAGGCGGTAATACGGT
TATCCACAGAATCAGGGGATAACGCAGGAAAGAACATGTGAGCAAAAGGCCAGCAAAAGGCCAGGAACCGTAAAAA
GGCCGCGTTGCTGGCGTTTTTCCATAGGCTCCGCCCCCCTGACGAGCATCACAAAAATCGACGCTCAAGTCAGAGG
TGGCGAAACCCGACAGGACTATAAAGATACCAGGCGTTTCCCCCTGGAAGCTCCCTCGTGCGCTCTCCTGTTCCGA
CCCTGCCGCTTACCGGATACCTGTCCGCCTTTCTCCCTTCGGGAAGCGTGGCGCTTTCTCAATGCTCACGCTGTAG
GTATCTCAGTTCGGTGTAGGTCGTTCGCTCCAAGCTGGGCTGTGTGCACGAACCCCCCGTTCAGCCCGACCGCTGC
GCCTTATCCGGTAACTATCGTCTTGAGTCCAACCCGGTAAGACACGACTTATCGCCACTGGCAGCAGCCACTGGTA
ACAGGATTAGCAGAGCGAGGTATGTAGGCGGTGCTACAGAGTTCTTGAAGTGGTGGCCTAACTACGGCTACACTAG

AAGGACAGTATTTGGTATCTGCGCTCTGCTGAAGCCAGTTACCTTCGGAAAAAGAGTTGGTAGCTCTTGATCCGGC
AAACAAACCACCGCTGGTAGCGGTGGTTTTTTTGTTTGCAAGCAGCAGATTACGCGCAGAAAAAAAGGATCTCAAG
AAGATCCTTTGATCTTTTCTACGGGGTCTGACGCTCAGTGGAACGAAAACTCACGTTAAGGGATTTTGGTCATGAG
ATTATCAAAAAGGATCTTCACCTAGATCCTTTTAAATTAAAAATGAAGTTTTAAATCAATCTAAAGTATATATGAG
TAAACTTGGTCTGACAGTTACCAATGCTTAATCAGTGAGGCACCTATCTCAGCGATCTGTCTATTTCGTTCATCCA
TAGTTGCCTGACTCCCCGTCGTGTAGATAACTACGATACGGGAGGGCTTACCATCTGGCCCCAGTGCTGCAATGAT
ACCGCGAGACCCACGCTCACCGGCTCCAGATTTATCAGCAATAAACCAGCCAGCCGGAAGGGCCGAGCGCAGAAGT
GGTCCTGCAACTTTATCCGCCTCCATCCAGTCTATTAATTGTTGCCGGGAAGCTAGAGTAAGTAGTTCGCCAGTTA
ATAGTTTGCGCAACGTTGTTGCCATTGCTACAGGCATCGTGGTGTCACGCTCGTCGTTTGGTATGGCTTCATTCAG
CTCCGGTTCCCAACGATCAAGGCGAGTTACATGATCCCCCATGTTGTGCAAAAAAGCGGTTAGCTCCTTCGGTCCT
CCGATCGTTGTCAGAAGTAAGTTGGCCGCAGTGTTATCACTCATGGTTATGGCAGCACTGCATAATTCTCTTACTG
TCATGCCATCCGTAAGATGCTTTTCTGTGACTGGTGAGTACTCAACCAAGTCATTCTGAGAATAGTGTATGCGGCG
ACCGAGTTGCTCTTGCCCGGCGTCAATACGGGATAATACCGCGCCACATAGCAGAACTTTAAAAGTGCTCATCATT
GGAAAACGTTCTTCGGGGCGAAAACTCTCAAGGATCTTACCGCTGTTGAGATCCAGTTCGATGTAACCCACTCGTG
CACCCAACTGATCTTCAGCATCTTTTACTTTCACCAGCGTTTCTGGGTGAGCAAAAACAGGAAGGCAAAATGCCGC
AAAAAAGGGAATAAGGGCGACACGGAAATGTTGAATACTCATACTCTTCCTTTTTCAATATTATTGAAGCATTTAT
CAGGGTTATTGTCTCATGAGCGGATACATATTTGAATGTATTTAGAAAAATAAACAAATAGGGGTTCCGCGCACAT
TTCCCCGAAAAGTGCCACCTGACGTCTAAGAAACCATTATTATCATGACATTAACCTATAAAAATAGGCGTATCAC
GAGGCCCTTTCGTC

pHygroCas9-U6gRNA – 20nt gRNA insertion, green highlight; U6 promoter, yellow highlight; poly(T)/poly(A), blue highlight; 3xFLAG-NLS-Cas9, gray highlight; Hygromycin resistance gene, pink highlight.

TCGCGCGTTTCGGTGATGACGGTGAAAACCTCTGACACATGCAGCTCCCGGAGACGGTCACAGCatctgttcgac
ttgcagcctgaaatacggcacgagtaggaaaagccgagtcaaatgccgaatgcagagtctcattacagc
acaatcaactcaagaaaaactcgacactttttttaccatttgcacttaaatcctttttttattcgttatgt
atacttttttttggtccctaaccaaaacaaaaccaaactctcttagtcgtgcctctatatttaaaactat
caatttattatagtcaataaatcgaactgtgttttcaacaaacgaacaataggacactttgattctaaa
ggaaattttgaaaatcttaagcagagggttcttaagaccatttgccaattcttataattctcaactgct
ctttcctgatgttgatcatttatataggtatgttttcctcaatacttcgXXXXXXXXXXXXXXXXXXX
gttttagagctagaaatagcaagttaaaataaggctagtccgttatcaacttgaaaaagtggcaccgag
tcggtgctttttttagatctggaaaaatgatgtgacagtggaaatgagaagtaggtgcatctgcaaagat
ttgattcagagttgatgccattcatgatttttttttagaagtatcatttgtgtttttagaatatagaat
taattcttgaagacgaaagggcctcgtgatacgccGCGGATGCCGGGAGCAGACAAGCCCGTCAGGGCGCGT
CAGCGGGTGTTGGCGGGTGTCGGGGCTGGCTTAACTATGCGGCATCAGAGCAGATTGTACTGAGAGTGCACCATAT
GCGGTGTGAAATACCGCACAGATGCGTAAGGAGAAAATACCGCATCAGGCGCCATTCGCCATTCAGGCTGCGCAAC
TGTTGGGAAGGGCGATCGGTGCGGGCCTCTTCGCTATTACGCCAGCTGGCGAAAGGGGGATGTGCTGCAAGGCGAT
TAAGTTGGGTAACGCCAGGGTTTTCCCAGTCACGACGTTGTAAAACGACGGCCAGTGCCAAGCTTCGTTGCAGGAC
AGGATGTGGTGCCCGATGTGACTAGCTCTTTGCTGCAGGCCGTCCTATCCTCTGGTTCCGATAAGAGACCCAGAAC
TCCGGCCCCCCACCGCCCACCGCCACCCCCATACATATGTGGTACGCAAGTAAGAGTGCCTGCGCATGCCCCATGT
GCCCCACCAAGAGTTTTGCATCCCATACAAGTCGCCAAAGTGGAGAACCGAACCAATTCTTCGCGGGCAGAACAAA
ACCTTCTGCACACGTCTCCACTCGAATTTGGAGCCGGCCGGCGTGTGCAAAAGAGGTGAATCGAACGAAAGACCCG
TGTGTAAAGCCGCGTTTCCAAAATGTATAAAACCGAGAGCATCTGGCCAATGTGCATCAGTTGTGGTCAGCAGCAA
AATCAAGTGAATCATCTCAGTGCAACTAAacaccATGGACTATAAGGACCACGACGGAGACTACAAGGATCATGAT
ATTGATTACAAAGACGATGACGATAAGGCCCCAAAGAAGAAGCGGAAGGTCGGTATCCACGGAGTCCCAGCAGCCG
ACAAGAAGTACAGCATCGGCCTGGACATCGGCACCAACTCTGTGGGCTGGGCCGTGATCACCGACGAGTACAAGGT
GCCCAGCAAGAAATTCAAGGTGCTGGGCAACACCGACCGGCACAGCATCAAGAAGAACCTGATCGGAGCCCTGCTG
TTCGACAGCGGCGAAACAGCCGAGGCCACCCGGCTGAAGAGAACCGCCAGAAGAAGATACACCAGACGGAAGAACC
GGATCTGCTATCTGCAAGAGATCTTCAGCAACGAGATGGCCAAGGTGGACGACAGCTTCTTCCACAGACTGGAAGA
GTCCTTCCTGGTGGAAGAGGATAAGAAGCACGAGCGGCACCCCATCTTCGGCAACATCGTGGACGAGGTGGCCTAC
CACGAGAAGTACCCCACCATCTACCACCTGAGAAAGAAACTGGTGGACAGCACCGACAAGGCCGACCTGCGGCTGA
TCTATCTGGCCCTGGCCCACATGATCAAGTTCCGGGGCCACTTCCTGATCGAGGGCGACCTGAACCCCGACAACAG
CGACGTGGACAAGCTGTTCATCCAGCTGGTGCAGACCTACAACCAGCTGTTCGAGGAAAACCCCATCAACGCCAGC
GGCGTGGACGCCAAGGCCATCCTGTCTGCCAGACTGAGCAAGAGCAGACGGCTGGAAAATCTGATCGCCCAGCTGC

CCGGCGAGAAGAAGAATGGCCTGTTCGGAAACCTGATTGCCCTGAGCCTGGGCCTGACCCCCAACTTCAAGAGCAA
CTTCGACCTGGCCGAGGATGCCAAACTGCAGCTGAGCAAGGACACCTACGACGACGACCTGGACAACCTGCTGGCC
CAGATCGGCGACCAGTACGCCGACCTGTTTCTGGCCGCCAAGAACCTGTCCGACGCCATCCTGCTGAGCGACATCC
TGAGAGTGAACACCGAGATCACCAAGGCCCCCCTGAGCGCCTCTATGATCAAGAGATACGACGAGCACCACCAGGA
CCTGACCCTGCTGAAAGCTCTCGTGCGGCAGCAGCTGCCTGAGAAGTACAAAGAGATTTTCTTCGACCAGAGCAAG
AACGGCTACGCCGGCTACATTGACGGCGGAGCCAGCCAGGAAGAGTTCTACAAGTTCATCAAGCCCATCCTGGAAA
AGATGGACGGCACCGAGGAACTGCTCGTGAAGCTGAACAGAGAGGACCTGCTGCGGAAGCAGCGGACCTTCGACAA
CGGCAGCATCCCCCACCAGATCCACCTGGGAGAGCTGCACGCCATTCTGCGGCGGCAGGAAGATTTTTACCCATTC
CTGAAGGACAACCGGGAAAAGATCGAGAAGATCCTGACCTTCCGCATCCCCTACTACGTGGGCCCTCTGGCCAGGG
GAAACAGCAGATTCGCCTGGATGACCAGAAAGAGCGAGGAAACCATCACCCCCTGGAACTTCGAGGAAGTGGTGGA
CAAGGGCGCTTCCGCCCAGAGCTTCATCGAGCGGATGACCAACTTCGATAAGAACCTGCCCAACGAGAAGGTGCTG
CCCAAGCACAGCCTGCTGTACGAGTACTTCACCGTGTATAACGAGCTGACCAAAGTGAAATACGTGACCGAGGGAA
TGAGAAAGCCCGCCTTCCTGAGCGGCGAGCAGAAAAAGGCCATCGTGGACCTGCTGTTCAAGACCAACCGGAAAGT
GACCGTGAAGCAGCTGAAAGAGGACTACTTCAAGAAAATCGAGTGCTTCGACTCCGTGGAAATCTCCGGCGTGGAA
GATCGGTTCAACGCCTCCCTGGGCACATACCACGATCTGCTGAAAATTATCAAGGACAAGGACTTCCTGGACAATG
AGGAAAACGAGGACATTCTGGAAGATATCGTGCTGACCCTGACACTGTTTGAGGACAGAGAGATGATCGAGGAACG
GCTGAAAACCTATGCCCACCTGTTCGACGACAAAGTGATGAAGCAGCTGAAGCGGCGGAGATACACCGGCTGGGGC
AGGCTGAGCCGGAAGCTGATCAACGGCATCCGGGACAAGCAGTCCGGCAAGACAATCCTGGATTTCCTGAAGTCCG
ACGGCTTCGCCAACAGAAACTTCATGCAGCTGATCCACGACGACAGCCTGACCTTTAAAGAGGACATCCAGAAAGC
CCAGGTGTCCGGCCAGGGCGATAGCCTGCACGAGCACATTGCCAATCTGGCCGGCAGCCCCGCCATTAAGAAGGGC
ATCCTGCAGACAGTGAAGGTGGTGGACGAGCTCGTGAAAGTGATGGGCCGGCACAAGCCCGAGAACATCGTGATCG
AAATGGCCAGAGAGAACCAGACCACCCAGAAGGGACAGAAGAACAGCCGCGAGAGAATGAAGCGGATCGAAGAGGG
CATCAAAGAGCTGGGCAGCCAGATCCTGAAAGAACACCCCGTGGAAAACACCCAGCTGCAGAACGAGAAGCTGTAC
CTGTACTACCTGCAGAATGGGCGGGATATGTACGTGGACCAGGAACTGGACATCAACCGGCTGTCCGACTACGATG
TGGACCATATCGTGCCTCAGAGCTTTCTGAAGGACGACTCCATCGACAACAAGGTGCTGACCAGAAGCGACAAGAA
CCGGGGCAAGAGCGACAACGTGCCCTCCGAAGAGGTCGTGAAGAAGATGAAGAACTACTGGCGGCAGCTGCTGAAC
GCCAAGCTGATTACCCAGAGAAAGTTCGACAATCTGACCAAGGCCGAGAGAGGCGGCCTGAGCGAACTGGATAAGG
CCGGCTTCATCAAGAGACAGCTGGTGGAAACCCGGCAGATCACAAAGCACGTGGCACAGATCCTGGACTCCCGGAT
GAACACTAAGTACGACGAGAATGACAAGCTGATCCGGGAAGTGAAAGTGATCACCCTGAAGTCCAAGCTGGTGTCC
GATTTCCGGAAGGATTTCCAGTTTTACAAAGTGCGCGAGATCAACAACTACCACCACGCCCACGACGCCTACCTGA
ACGCCGTCGTGGGAACCGCCCTGATCAAAAAGTACCCTAAGCTGGAAAGCGAGTTCGTGTACGGCGACTACAAGGT
GTACGACGTGCGGAAGATGATCGCCAAGAGCGAGCAGGAAATCGGCAAGGCTACCGCCAAGTACTTCTTCTACAGC
AACATCATGAACTTTTTCAAGACCGAGATTACCCTGGCCAACGGCGAGATCCGGAAGCGGCCTCTGATCGAGACAA
ACGGCGAAACCGGGGAGATCGTGTGGGATAAGGGCCGGGATTTTGCCACCGTGCGGAAAGTGCTGAGCATGCCCCA
AGTGAATATCGTGAAAAAGACCGAGGTGCAGACAGGCGGCTTCAGCAAAGAGTCTATCCTGCCCAAGAGGAACAGC
GATAAGCTGATCGCCAGAAAGAAGGACTGGGACCCTAAGAAGTACGGCGGCTTCGACAGCCCCACCGTGGCCTATT
CTGTGCTGGTGGTGGCCAAAGTGGAAAAGGGCAAGTCCAAGAAACTGAAGAGTGTGAAAGAGCTGCTGGGGATCAC
CATCATGGAAAGAAGCAGCTTCGAGAAGAATCCCATCGACTTTCTGGAAGCCAAGGGCTACAAAGAAGTGAAAAAG
GACCTGATCATCAAGCTGCCTAAGTACTCCCTGTTCGAGCTGGAAAACGGCCGGAAGAGAATGCTGGCCTCTGCCG
GCGAACTGCAGAAGGGAAACGAACTGGCCCTGCCCTCCAAATATGTGAACTTCCTGTACCTGGCCAGCCACTATGA
GAAGCTGAAGGGCTCCCCCGAGGATAATGAGCAGAAACAGCTGTTTGTGGAACAGCACAAGCACTACCTGGACGAG
ATCATCGAGCAGATCAGCGAGTTCTCCAAGAGAGTGATCCTGGCCGACGCTAATCTGGACAAAGTGCTGTCCGCCT
ACAACAAGCACCGGGATAAGCCCATCAGAGAGCAGGCCGAGAATATCATCCACCTGTTTACCCTGACCAATCTGGG
AGCCCCTGCCGCCTTCAAGTACTTTGACACCACCATCGACCGGAAGAGGTACACCAGCACCAAAGAGGTGCTGGAC
GCCACCCTGATCCACCAGAGCATCACCGGCCTGTACGAGACACGGATCGACCTGTCTCAGCTGGGAGGCGACtgaG
TTTAAACCCGCTGATCAGCCTCGACTGTGCCTTCTAAGATCCAGACATGATAAGATACATTGATGAGTTTGGACAA
ACCACAACTAGAATGCAGTGAAAAAAATGCTTTATTTGTGAAATTTGTGATGCTATTGCTTTATTTGTAACCATTA
TAAGCTGCAATAAACAAGTTAACAACAACAAAAGCTTGCATGCCTGCAGGTCGACTCTAGAGGATCCGGTGCGTGG
TGGTTTCATGCTTCTGGGAACGGCAAATGGGTTTAGGATTGGGAACCCCTCATCATCTGTTGGAATATACTATTCA
ACCTACAAAAATAACGTTAAACAACACTACTTTATATTTGATATGAATGGCCACACCTTTTATGCCATAAAACATA
TTGTAAGAGAATACCACTCTTTTTATTCCTTCTTTCCTTCTTGTACGTTTTTTGCTGTGAGTAGGTCGTGGTGCTG
GTGTTGCAGTTGAAATAACTTAAAATATAAATCATAAAACTCAAACATAAACTTGACTATTTATTTATTTATTAAG
AAAGGAAATATAAATTATAAATTACAACAGGTTATGGGGATCCCCAGCTTGGCCACCATGGAAAGCCTGAGCGTA
CCGCGACGTCTGTCGAGAAGTTTCTGATCGAAAAGTTCGACAGCGTCTCCGACCTGATGCAGCTCTCGGAGGGCGA
AGAATCTCGTGCTTTCAGCTTCGATGTAGGAGGGCGTGGATATGTCCTGCGGGTAAATAGCTGCGGCGATGGTTTC
TACAAAGATCGTTATGTTTATCGGCACTTTGCATCGGCCGCGCTCCCGATTCCGGAAGTGCTTGACATTGGGGAAT
TCAGCGAGAGCCTGACCTATTGCATCTCCCGCCGTGCACAGGGTGTCACGTTGCAAGACCTGCCTGAAACCGAACT

GCCCGCTGTTCTGCAGCCGGTCGCGGAGGCCATGGATGCGATCGCTGCGGCCGATCTTAGCCAGACGAGCGGGTTC
GGCCCATTCGGACCGCAAGGAATCGGTCAATACACTACATGGCGTGATTTCATATGCGCGATTGCTGATCCCCATG
TGTATCACTGGCAAACTGTGATGGACGACACCGTCAGTGCGTCCGTCGCGCAGGCTCTCGATGAGCTGATGCTTTG
GGCCGAGGACTGCCCCGAAGTCCGGCACCTCGTGCACGCGGATTTCGGCTCCAACAATGTCCTGACGGACAATGGC
CGCATAACAGCGGTCATTGACTGGAGCGAGGCGATGTTCGGGGATTCCCAATACGAGGTCGCCAACATCTTCTTCT
GGAGGCCGTGGTTGGCTTGTATGGAGCAGCAGACGCGCTACTTCGAGCGGAGGCATCCGGAGCTTGCAGGATCGCC
GCGGCTCCGGGCGTATATGCTCCGCATTGGTCTTGACCAACTCTATCAGAGCTTGGTTGACGGCAATTTCGATGAT
GCAGCTTGGGCGCAGGGTCGATGCGACGCAATCGTCCGATCCGGAGCCGGGACTGTCGGGCGTACACAAATCGCCC
GCAGAAGCGCGGCCGTCTGGACCGATGGCTGTGTAGAAGTACTCGCCGATAGTGGAAACCGACGCCCCAGCACTCG
TCCGAGGGCAAAGGAATAGAGTAGATGCCGACCGAACAAGAGCTGATTTCGAGAACGCCTCAGCCAGCAACTCGCG
CGAGCCTAGCAAGGCAAATGCGAGAGAACGGCCTTACGCTTGGTGGCACAGTTCTCGTCCACAGTTCGCTAAGCTC
GCTCGGCTGGGTCGCGGGAGGGCCGGTCGCAGTGATTCAGGCCCTTCTGGATTGTGTTGGTCCCCAGGGCACGATT
GTCATGCCCACGCACTCGGGTGATCTGACTGATCCCGCAGATTGGAGATCGCCGCCCGTGCCTGCCGATTGGGTGC
AGATCAGCCTCGAGGCCAGCTAGCTTGAACTTGTTTATTGCAGCTTATAATGGTTACAAATAAAGCAATAGCATCA
CAAATTTCACAAATAAAGCATTTTTTTCACTGCATTCTAGTTGTGGTTTGTCCAAACTCATCAATGTATCTTATCA
TGTCTGGATCGGGTACCGAGCTCGAATTCGTAATCATGTCATAGCTGTTTCCTGTGTGAAATTGTTATCCGCTCAC
AATTCCACACAACATACGAGCCGGAAGCATAAAGTGTAAAGCCTGGGGTGCCTAATGAGTGAGCTAACTCACATTA
ATTGCGTTGCGCTCACTGCCCGCTTTCCAGTCGGGAAACCTGTCGTGCCAGCTGCATTAATGAATCGGCCAACGCG
CGGGGAGAGGCGGTTTGCGTATTGGGCGCTCTTCCGCTTCCTCGCTCACTGACTCGCTGCGCTCGGTCGTTCGGCT
GCGGCGAGCGGTATCAGCTCACTCAAAGGCGGTAATACGGTTATCCACAGAATCAGGGGATAACGCAGGAAAGAAC
ATGTGAGCAAAAGGCCAGCAAAAGGCCAGGAACCGTAAAAAGGCCGCGTTGCTGGCGTTTTTCCATAGGCTCCGCC
CCCCTGACGAGCATCACAAAAATCGACGCTCAAGTCAGAGGTGGCGAAACCCGACAGGACTATAAAGATACCAGGC
GTTTCCCCCTGGAAGCTCCCTCGTGCGCTCTCCTGTTCCGACCCTGCCGCTTACCGGATACCTGTCCGCCTTTCTC
CCTTCGGGAAGCGTGGCGCTTTCTCAATGCTCACGCTGTAGGTATCTCAGTTCGGTGTAGGTCGTTCGCTCCAAGC
TGGGCTGTGTGCACGAACCCCCCGTTCAGCCCGACCGCTGCGCCTTATCCGGTAACTATCGTCTTGAGTCCAACCC
GGTAAGACACGACTTATCGCCACTGGCAGCAGCCACTGGTAACAGGATTAGCAGAGCGAGGTATGTAGGCGGTGCT
ACAGAGTTCTTGAAGTGGTGGCCTAACTACGGCTACACTAGAAGGACAGTATTTGGTATCTGCGCTCTGCTGAAGC
CAGTTACCTTCGGAAAAAGAGTTGGTAGCTCTTGATCCGGCAAACAAACCACCGCTGGTAGCGGTGGTTTTTTTGT
TTGCAAGCAGCAGATTACGCGCAGAAAAAAAGGATCTCAAGAAGATCCTTTGATCTTTTCTACGGGGTCTGACGCT
CAGTGGAACGAAAACTCACGTTAAGGGATTTTGGTCATGAGATTATCAAAAAGGATCTTCACCTAGATCCTTTTAA
ATTAAAAATGAAGTTTTAAATCAATCTAAAGTATATATGAGTAAACTTGGTCTGACAGTTACCAATGCTTAATCAG
TGAGGCACCTATCTCAGCGATCTGTCTATTTCGTTCATCCATAGTTGCCTGACTCCCCGTCGTGTAGATAACTACG
ATACGGGAGGGCTTACCATCTGGCCCCAGTGCTGCAATGATACCGCGAGACCCACGCTCACCGGCTCCAGATTTAT
CAGCAATAAACCAGCCAGCCGGAAGGGCCGAGCGCAGAAGTGGTCCTGCAACTTTATCCGCCTCCATCCAGTCTAT
TAATTGTTGCCGGGAAGCTAGAGTAAGTAGTTCGCCAGTTAATAGTTTGCGCAACGTTGTTGCCATTGCTACAGGC
ATCGTGGTGTCACGCTCGTCGTTTGGTATGGCTTCATTCAGCTCCGGTTCCCAACGATCAAGGCGAGTTACATGAT
CCCCCATGTTGTGCAAAAAAGCGGTTAGCTCCTTCGGTCCTCCGATCGTTGTCAGAAGTAAGTTGGCCGCAGTGTT
ATCACTCATGGTTATGGCAGCACTGCATAATTCTCTTACTGTCATGCCATCCGTAAGATGCTTTTCTGTGACTGGT
GAGTACTCAACCAAGTCATTCTGAGAATAGTGTATGCGGCGACCGAGTTGCTCTTGCCCGGCGTCAATACGGGATA
ATACCGCGCCACATAGCAGAACTTTAAAAGTGCTCATCATTGGAAAACGTTCTTCGGGGCGAAAACTCTCAAGGAT
CTTACCGCTGTTGAGATCCAGTTCGATGTAACCCACTCGTGCACCCAACTGATCTTCAGCATCTTTTACTTTCACC
AGCGTTTCTGGGTGAGCAAAAACAGGAAGGCAAAATGCCGCAAAAAAGGGAATAAGGGCGACACGGAAATGTTGAA
TACTCATACTCTTCCTTTTTCAATATTATTGAAGCATTTATCAGGGTTATTGTCTCATGAGCGGATACATATTTGA
ATGTATTTAGAAAAATAAACAAATAGGGGTTCCGCGCACATTTCCCCGAAAAGTGCCACCTGACGTCTAAGAAACC
ATTATTATCATGACATTAACCTATAAAAATAGGCGTATCACGAGGCCCTTTCGTC
